# A meta-analysis of genetic estimates for economically important traits in ducks

**DOI:** 10.1016/j.vas.2024.100405

**Published:** 2024-11-01

**Authors:** Navid Ghavi Hossein-Zadeh

**Affiliations:** Department of Animal Science, Faculty of Agricultural Sciences, University of Guilan, Rasht, 41635-1314, Iran

**Keywords:** Additive genetic variation, Duck, Egg production, Feed intake, Systematic review

## Abstract

This study aimed to gather published genetic parameter estimates for economically important traits in ducks through a meta-analysis utilizing the random-effects model. The present study used a dataset on genetic parameters of various duck traits, including 275 genetic correlation estimates and 233 heritability estimates from 31 studies published between 1988 and 2024. The heritability estimates for growth traits were generally low to high and varied from 0.154 (for body weight gain) to 0.405 (for body weight at first egg), respectively. Results showed that heritability estimates for egg production and quality traits were generally low to moderate, ranging from 0.119 (for egg shell strength) to 0.340 (for egg weight). The heritability estimates for feeding traits were generally moderate to high and varied from 0.266 (for residual feed intake) to 0.624 (for meal feed intake), respectively. The results indicate that there was a high genetic correlation (0.827, P < 0.05) between feed intake and residual feed intake, but low to moderate genetic correlations (P < 0.05) were found between feed intake and feed conversion ratio (0.318). The results of the current meta-analysis supported the hypothesis that these duck traits exhibit additive genetic variation. Genetic selection schemes for ducks may thus potentially take advantage of the available additive genetic variation in these traits. Furthermore, in cases where accurate estimates for economically significant traits in duck populations across the globe are unavailable, the average genetic parameter estimates presented in this meta-analysis can be used in breeding plans.

## 1 Introduction

A significant global consumer base, primarily in Asia and Europe, makes domestic ducks a valuable poultry. Duck meat is generally regarded as having a flavorful taste, being high in polyunsaturated fatty acids and amino acids, and having a relatively low fat content. Duck meat has been produced and consumed in considerable quantities worldwide in recent years. Breeders believe that body weight composition (e.g., breast muscle thickness and weight gain) is the most significant characteristic of ducks (Szwaczkowski et al., 2010). Increasing the size and proportion of key carcass segments, especially the breast muscle, is critical to the profitability of the duck industry (Cai et al., 2024; He et al., 2011). Duck meat has higher muscle fiber content and lipids (Chartrin et al., 2006; Smith et al., 1993). These properties reduce the ability to retain water and, unlike chicken meat, increase cooking loss (Cai et al., 2023). Some duck by-products, such as feet, neck, liver, and gizzards, were also popular. Meat quality characteristics directly impact consumers' purchasing decisions as they become more selective and seek higher quality products (Zhang et al., 2022). Furthermore, eggs are considered an excellent source of nutrients for humans as they contain sufficient amounts of proteins, fatty acids, vitamins, minerals and other nutrients for human daily needs (Sunwoo and Gujral, 2015). The internal quality of eggs influences both the consumers' health and their edibility (Nys et al., 2011; Sharaf Eddin et al., 2019). Numerous variables, such as the laying ducks' nutritional status, egg storage duration and condition, can directly impact the characteristics of egg quality (Liu et al., 2021a; Pires et al., 2020; Rathnayaka et al., 2020; Saleh et al., 2020). Moreover, genetics is another important component (Liu et al., 2018).

The main methods currently used in duck breeding are traditional phenotypic selection and pedigree analysis, followed by assessment of estimated breeding values ​​(EBVs) using the BLUP approach. Using phenotypic and pedigree data, numerous attempts have been made to enhance the productive traits of ducks in duck breeding programs (Xu et al., 2018). Duck carcass traits are more expensive to measure and are primarily recorded after the birds are slaughtered, despite the fact that selecting for productive traits in ducks is feasible. Furthermore, sib-testing is a common procedure used in traditional selection for these traits that cannot be measured in vivo; the only variation to be used is within-family variation, which limits the accuracy of the selection process. The improvement of carcass composition traits is not given much attention in the current duck breeding programs for the reasons mentioned above (Chen et al., 2021). Furthermore, the use of genomic prediction in ducks has not received much attention up until now (Cai et al., 2023, 2024; Zhang et al., 2022).

The parameters associated with economic traits should be carefully calculated and an in-depth study of their genetic characteristics should be carried out before creating a breeding program (Gholipour et al., 2022). Thus, precisely estimating the genetic parameters of economically significant traits is one of the best ways to enhance the genetics of these populations in breeding programs and to raise the production capacity of the duck population. To support the advancement of duck breeding and the duck industry, it is necessary to identify the genetic architecture underlying economically important traits in ducks. Numerous studies have been conducted in the last few decades to estimate genetic parameters for various traits of interest in ducks using various statistical models and methods. The sample size of these studies varies, and the results can occasionally be contradictory. The results of individual studies may have been measured with a particular error, leading to uncertainty about estimates and potentially affecting breeding decisions and programs. The accuracy of breeding value estimates depends on the accurate estimation of genetic parameters, and the application of novel, scientific techniques like meta-analysis is one of the most efficient ways to improve estimate accuracy. Reducing the unnecessary repetition of animal experiments and producing more accurate estimates are two benefits of combining results through meta-analysis (Hooijmans et al., 2014). Furthermore, due to the more exploratory nature of animal experiments compared to clinical studies, meta-analyzes of animal studies have a higher chance of identifying plausible sources of heterogeneity (Sena et al., 2010). Consequently, a meta-analysis is a technique that generates a report with a single result and increased statistical power by combining various individual and independent results from studies that have a common field (Littell et al., 2008). In order to obtain a single range of estimates using meta-analysis, which will be helpful in breeding programs, it is necessary to take into account the variability of the scope of genetic parameter estimates as well as the potential for errors in the literature and published articles.

To the author's knowledge, no specific meta-analysis of genetic parameters for growth, reproduction, carcass, feeding and egg production and quality traits of ducks has been reported in the literature. Therefore, the aim of this study was to conduct a meta-analysis using a random-effects model to bring together various published genetic parameter estimates for the above traits in ducks. The results aim to support more efficient breeding programs by providing a reference for genetic parameters for which precise estimates are limited or unavailable. This meta-analysis provides fundamental data that breeders can use when selecting important traits, allowing them to use additive genetic variation to improve economic traits in duck populations worldwide.

## 2 Materials and methods

### 2.1 Gathering required information and inclusion criteria

**Fig. 1:**
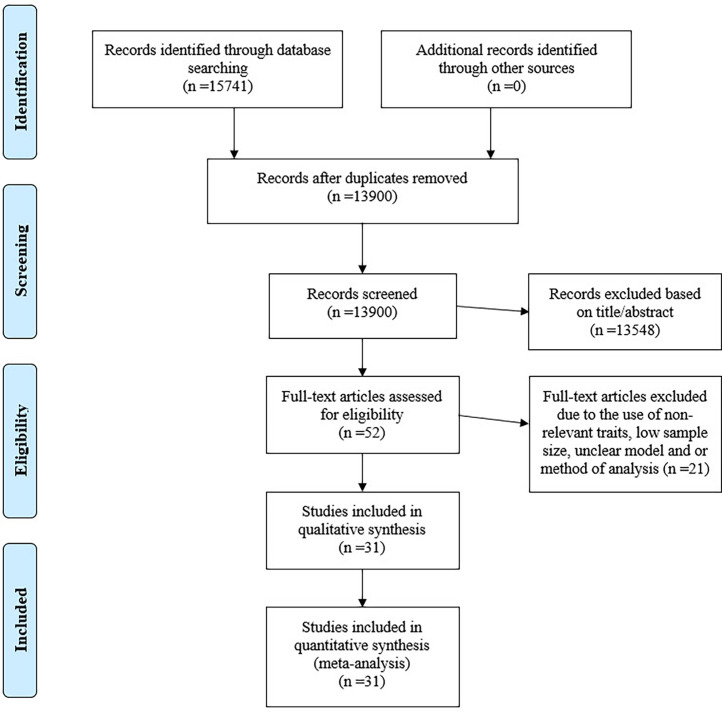
A flow diagram representing the chosen studies incorporated into the meta-analysis using the PRISMA approach.

This work followed the PRISMA (Preferred Reporting Items for Systematic Reviews and Meta-Analyzes) guideline for handling meta-analyzes and systematic reviews (Fig. 1) (Moher et al., 2009). A systematic literature search was carried out in Google Scholar (https://scholar.google.com), ResearchGate (https://www.researchgate.net), NCBI (https://www.ncbi.nlm.nih.gov), and ISI Web of Knowledge (https://apps.webofknowledge.com) databases to identify studies that reported genetic parameter estimates for economically important traits in ducks. To align with PRISMA guidelines, the PICO framework was used to guide the literature search strategy as follows: Population (P): Ducks with economically important traits; Intervention (I): Studies reporting genetic parameter estimates for these traits; Comparator (C): Genetic parameters reported across different studies and models; Outcome (O): Heritability and genetic correlation estimates for traits, allowing for synthesis and meta-analysis. The search terms used were carefully selected to cover all relevant studies and included combinations of the following keywords: ‘genetic parameters’ AND ‘economically important traits’ AND (‘growth traits’ OR ‘production traits’ OR ‘egg production’ OR ‘egg quality traits’ OR ‘meat quality’ OR ‘carcass traits’ OR ‘reproductive traits’) AND ‘duck’ AND (‘heritability’ OR ‘genetic correlation’ OR ‘genetic evaluation’). The use of these terms with Boolean operators enabled a comprehensive yet targeted search aimed at retrieving studies directly related to genetic evaluations in ducks. Despite efforts to refine the search, the initial search yielded 15741 articles, likely due to the use of broad terms to capture studies across multiple economically important duck traits. Initial screening by title and abstract allowed us to filter this set to 52 studies deemed suitable for full-text review, which, although broad, ensured thorough coverage of the literature. It was recognized that this approach produced a lower hit rate than typical systematic reviews, but it was necessary to avoid omitting relevant studies on under-researched traits. The final dataset comprised 275 genetic correlation estimates and 233 heritability estimates from 31 studies (Table 1), covering studies published between 1988 and 2024. Only research articles published in indexed journals and conference proceedings were included. Citations within relevant papers were also reviewed to identify additional studies. However, no further studies meeting the inclusion criteria were found through this supplementary search process. Techniques used in the studies included animal and/or reduced animal models, least-squares, analysis of variance (ANOVA), and restricted maximum likelihood (REML) for variance component estimation. To avoid duplicate data, only the most recent publication was included if estimates appeared in multiple sources. Additionally, only traits with at least three estimates from different studies were included.

**Table 1:** The characteristics of studies included in the database for conducting this meta-analysis.

Reference	Country/Region	Breed	Analysis method	Analysis model
Basso et al. (2012)	France	INRA I444 and I37	REML	Animal
Boonlert et al. (2005)	Philippine	Philippine Mallard	REML	Sire+Dam
Brun and Larzul (2003)	France	Muscovy	REML	Animal
Cai et al. (2023)	China	Pekin*Mallard	REML	Animal
Cai et al. (2024)	China	Pekin*Mallard	REML	Animal
Chapuis et al. (2022)	France	White Pekin	REML	Animal
Cheng et al. (1995)	Taiwan	Brown Tsaiya	REML	Animal
Damayanti et al. (2019)	Indonesia	Alabio	ANOVA	Sire
Drouilhet et al. (2014)	France	Muscovy	REML	Sire+Dam
Duggan et al. (2017)	UK	Pekin	REML	Animal
El-Deghadi et al. (2022)	Egypt	Domyati and Khaki-Campbell	REML	Sire
El-Sayiad et al. (1988)	Egypt	Peking	Least-squares	Sire+Dam
Hu et al. (1999)	Taiwan	Muscovy	REML	Animal
Hu et al. (2004)	Taiwan	Muscovy	REML	Animal
Lazrul (2002)	France	Mule duck	REML	Sire+Dam
Li et al. (2020)	China	Pekin	REML	Animal
Lin et al. (2016)	China	Shan Ma	REML	Animal
Liu et al. (2021a)	China	Pekin*Mallard	REML	Animal
Liu et al. (2021b)	China	Pekin*Mallard	REML	Animal
Marie-Etancelin et al. (2011)	France	Common and Muscovy lines	REML	Sire
Mucha et al. (2014)	Poland	A-55 and GL-30 crosses	REML	Animal
Padhi et al. (2022)	India	Kuzi	Least-squares	Sire
Qiangqiang et al. (2002)	China	Line A	REML	Animal
Szwaczkowski et al. (2010)	Poland	Pekin	REML	Animal
Tai et al. (1989)	Taiwan	Brown Tsaiya	REML	Sire+Dam
Wähner et al. (2017)	China	Pekin	REML	Animal
Xu et al. (2011)	China	Pekin	REML	Animal
Xu et al. (2018)	China	Pekin	REML	Animal
Zeng et al. (2018)	China	Shaoxing and Jinyun	REML	Animal
Zhang et al. (2017)	China	Pekin	REML	Animal
Zhang et al. (2022)	China	Pekin	REML	Animal

REML: Restricted maximum likelihood; ANOVA: Analysis of variance

Thirty economically important duck traits were examined and were grouped as follows: growth traits [body weight (BW), body weight at first egg (BWFE), and body weight gain (BWG, in g (BWG_g_) and g/day (BWG_gd_))], carcass traits [carcass weight (CW), liver weight (LW), breast muscle weight (BMW), breast muscle percentage (BMP), breast muscle thickness (BMT), abdominal fat weight (AFW), abdominal fat percentage (AFP), skin fat weight (SFW), skin fat percentage (SFP), and leg muscle weight (LMW) ], feeding traits [feed intake (FI, in g (FI_g_) and g/day (FI_gd_), residual feed intake (RFI), daily feeding rate (DFR), number of meals per day (NMD), meal feed intake (MFI), meal duration (MD), and feed conversion ratio (FCR)], egg production and quality traits [egg number (EN), egg weight (EW), egg shell strength (ESS), and egg mass (EM, in g (EM_g_) and g/day (EM_gd_))], and reproduction traits [fertility rate (FER), hatchability rate (HR), and age at first egg (AFE)]. Direct heritability estimates, genetic correlations between various traits, and their standard errors were among the data needed for the meta-analysis. The data structure information encompassed various aspects such as the breed, number of data collection years, publication year, journal name, country of origin, number of records, estimation method, analysis model, phenotypic mean, and standard deviation. The mean and standard deviations were computed for each trait using sample sizes as weights. To determine the total number of records for each phenotypic trait, the records from each article reporting the trait were added together. The calculation of the coefficient of variation in percentage (CV_i_(%)) for each i^th^ trait was as follows:

$$CV_{i}\left(\%\right)=\frac{s_{i}}{{\bar{X}}_{i}}\times 100$$

where *s_i_ =* the standard deviation of the *i*^th^ trait and ${\bar{X}}_{i}$= the mean of the trait.

Using the combined-variance method, approximate standard errors were derived for papers that did not report standard errors of genetic parameter estimates as follows (Sutton et al., 2000):

$$SE_{ij}=\sqrt{\frac{\left(\frac{\sum_{k=1}^{K}s_{ik}^{2}n_{ik}^{2}}{\sum_{k=1}^{K}n_{ik}}\right)}{{n^{\prime }}_{ij}}}$$

where SE_ij_ = the standard error predicted for the published genetic parameter estimate of the *i*^th^ trait in the *j*^th^ paper that did not report a standard error, *s*_ik_ = the published standard error of the genetic parameter estimate for the *i*^th^ trait in the *k*^th^ paper that reported the standard error, *n*_ik_ = the number of records that were utilized to predict the genetic parameter estimate that was published for the i^th^ trait in the k^th^ paper that reported the standard error, and *n´*_ij_ = the number of records that were utilized to predict the genetic parameter estimate that was published for the i^th^ trait in the j^th^ paper that did not report the standard error.

The published correlation estimates themselves are typically not used in meta-analyzes because their distribution is usually non-normal. Rather, all analyzes are performed using the converted values obtained from the transformation of the published correlations into the Fisher's Z scale. Converting the estimated parameters and their confidence intervals back into correlations would be necessary for presentation purposes (Borenstein et al., 2009). Fisher's Z transformation (Borenstein et al., 2009; Steel and Torrie, 1960) offers the following approximation of the normal scale:

$$Z_{ij}=0.5\left[\ln\left(1+r_{g_{ij}}\right)-\ln\left(1-r_{g_{ij}}\right)\right]$$

where r_gij_ = the published genetic correlation estimate for the *i*^th^ trait in the *j*^th^ paper. The equation that follows (Borenstein et al., 2009) was utilized in order to return to the initial scale:

$$r_{g_{ij}}^{*}=\frac{e^{2Z_{ij}}-1}{e^{2Z_{ij}}+1}$$

where $r_{g_{ij}}^{*}$= the re-transformed genetic correlation estimate for the *i*^th^ trait in the *j*^th^ paper is, while *Z*_ij_= the Fisher's *Z* transformation.

### 2.2 Meta-analysis model

In order to run a meta-analysis model with random-effects (Borenstein et al., 2009), the Comprehensive Meta-Analysis (CMA) software version 2.2 (Biostat, USA) was used to calculate the effect sizes for genetic parameter estimates. Variations in study results are observed in the random-effects model due to random changes in values and the possibility of repeated sampling. The general form of the random-effects model was as follows:

$${\hat{\theta }}_{i}=\bar{\theta }+u_{i}+e_{i}$$

where ${\hat{\theta }}_{i}$= parameter estimate published in the *i*^th^ paper, $\bar{\theta }$= the weighted average of the population parameters, u_i_= the component of deviation from the mean between studies, assumed as $u_{i}∼N\left(0,\tau^{2}\right)$, where $\tau^{2}$= variance, which represents the degree of heterogeneity between studies, e_i_= the within-study component due to sampling error in the parameter estimate in the *i*^th^ paper, assumed as $e_{i}∼N\left(0,\sigma_{e}^{2}\right)$, where $\sigma_{e}^{2}$is the within-study variance.

To show the effect size for each study, forest plots were made. The average heritability estimates for the traits under study or the genetic correlation estimates between traits with 95% confidence intervals were used to calculate the effect sizes for forest plots.

### 2.3 Assessment of heterogeneity

The I^2^ statistic was calculated and a chi-square (Q) test was run to evaluate heterogeneity. The Q test was utilized to assess the variation among study levels. Given the small number of included studies, the power of the Q test is approximately small, so the level of significance was set at 0.1 (Lean et al., 2009). Heterogeneity can be ascertained using the Q test, but it can also be computed using the I^2^ statistic (Lean et al., 2009):

$$I^{2}\left(\%\right)=\frac{Q-\left(k-1\right)}{Q}\times 100$$

where k = the number of studies and Q = the χ^2^ heterogeneity statistic and can be measured as follows:

$$Q={\sum_{i=1}^{k}w_{i}\left({\hat{\theta }}_{i}-\bar{\theta }\right)}^{2}$$

where *w*_i_ = the weight of parameter estimate (is considered the inverse of the published sampling variance for the parameter, $\frac{1}{s_{i}^{2}}$) in the *i*^th^ paper and k *=*the number of used papers. The percentage of variation between studies due to heterogeneity is represented by the I^2^ statistic. It is assumed that negative I^2^ values equal zero. I^2^ thus varies between 0 % and 100 %. (Lean et al., 2009). If it falls between 0 % and 40 %, its value might be insignificant. On the other hand, moderate levels of heterogeneity are generally represented by values between 40 and 60 %, and high levels of heterogeneity are represented by values between 60 and 100 %. The following formula would be used to get the lower and upper 95 % bounds of the estimated parameters for each trait:

$$LL_{\bar{\theta }}=\bar{\theta }-1.96\times SE_{\bar{\theta }}\begin{array}{cc} & and\begin{array}{cc} & UL_{\bar{\theta }}=\bar{\theta }+1.96\times SE_{\bar{\theta }} \end{array} \end{array}$$

where $SE_{\bar{\theta }}$= the predicted standard error for the estimated parameter $\bar{\theta }$, calculated by the following formula:

$$SE_{\bar{\theta }}=\sqrt{\frac{1}{\sum_{i=1}^{k}w_{i}}}$$

### 2.4 Evaluation of publication bias

To determine whether publication bias existed, Egger's linear regression asymmetry was applied. A significant publication bias was declared at P < 0.10. Also, to ascertain the number of missing studies, a trim-and-fill technique (Duval and Tweedie, 2000) was applied. Asymmetry is illustrated with funnel plots. The effect sizes in this plot are distributed symmetrically around the true effect size. The fact that there is no publication bias indicates that the most extreme estimates were not published. The weighted average effect size and its variance are recalculated with the estimated missing values included after the missing observations have been estimated. It is not appropriate to test for the possibility of publication bias if heterogeneity (Q test, P < 0.10) was discovered in the analyzed parameters, as this could lead to false-positive decisions (Ioannidis and Trikalinos, 2007).

## 3 Results

### 3.1 Summary statistics

Descriptive statistics of economically important traits in ducks are presented in Table 2. The results showed that growth traits had moderate to high weighted coefficients of variation, ranging from 35.68 (for BWFE) to 58.02 % (for BW). The weighted coefficients of variation for feeding traits were low to high and varied from 6.74 (for FI_g_) to 70.70 % (for MD). Among the egg production and quality traits, ESS and EN had the lowest (4.08 %) and highest (67.24 %) weighted coefficient of variation, respectively. Furthermore, among the reproductive traits, HR and AFE had the lowest (0.82 %) and highest (34.93 %) weighted coefficients of variation, respectively. In addition, the carcass traits had low to high weighted coefficients of variation, varying between 4.67 (for AFP) and 79.62 % (for AFW) (Table 2).

**Table 2:** Number of literature estimates (N), measurement units (Unit), the total number of records (Records), weighted mean (Mean), standard deviation (SD), and coefficient of variation (CV) for economically important traits in ducks.

Trait	Unit	Abbreviation	N	Records	Mean	SD	CV (%)
Feed intake	g	FI_g_	3	7944	5537.80	373.21	6.74
Feed intake	g/d	FI_gd_	7	10320	239.94	43.75	18.23
Residual feed intake	-	RFI	6	12310	0	-	-
Daily feeding rate	g/min	DFR	3	6794	23.79	4.55	19.13
Number of meals per day	-	NMD	3	6794	8.62	2.10	24.36
Meal feed intake	g	MFI	3	6794	38.70	7.86	20.31
Meal duration	s	MD	3	6794	115.32	81.53	70.70
Feed conversion ratio	-	FCR	10	18825	2.54	0.44	17.32
Body weight	g	BW	69	111004	2107.86	1223.03	58.02
Body weight at first egg	g	BWFE	5	1602	731.36	2049.98	35.68
Body weight gain	g	BWG_g_	4	10034	1884.87	862.41	45.75
Body weight gain	g/d	BWG_gd_	9	10406	71.07	32.81	46.17
Egg number	-	EN	21	23182	100.13	67.33	67.24
Egg weight	g	EW	14	16092	65.46	5.27	8.05
Egg shell strength	kg/cm^2^	ESS	3	3479	3.68	0.15	4.08
Egg mass	g	EM_g_	3	7283	3235.65	250.47	7.74
Egg mass	g/d	EM_gd_	3	3169	64.05	4.97	7.76
Fertility rate	%	FER	3	1945	73.67	9.40	12.76
Hatchability rate	%	HR	3	1935	71.01	0.58	0.82
Age at first egg	day	AFE	7	7340	158.82	55.47	34.93
Carcass weight	g	CW	3	4348	1998.72	509.82	25.51
Liver weight	g	LW	4	6674	428.68	196.63	45.87
Breast muscle weight	g	BMW	7	19752	194.58	80.76	41.50
Breast muscle percentage	%	BMP	5	17724	7.56	1.86	24.60
Breast muscle thickness	cm	BMT	7	26714	1.58	0.26	16.46
Abdominal fat weight	g	AFW	7	6014	74.94	59.67	79.62
Abdominal fat percentage	%	AFP	4	4151	2.14	0.10	4.67
Leg muscle weight	g	LMW	4	4735	207.93	140.86	67.74
Skin fat weight	g	SFW	3	2166	589.87	201.02	34.08
Skin fat percentage	%	SFP	3	2166	26.98	5.26	19.50

### 3.2 Heritability estimates

The effect sizes and heterogeneity of heritability estimates for economically important traits in ducks derived from the random-effects meta-analysis model are presented in Table 3. The heritability estimates for growth traits varied from low to high [from 0.154 (for BWG_g_) to 0.405 (for BWFE)]. For both BW and BWFE, the heritability estimates' standard errors were low, and their 95 % confidence intervals were fairly narrow Furthermore, heritability estimates were also significant for all growth traits (P < 0.05). Heritability estimates for growth traits were subjected to a heterogeneity test using the Q statistic, which revealed non-significant heterogeneity and a low Q value for the heritability estimate for BWFE (P > 0.10). However, the average heritability estimates for BW, BWG_g_, and BWG_gd_ experienced significant heterogeneities (P < 0.10). In agreement with the results observed by Q statistic, the I^2^ values showed minor heterogeneity for the heritability estimate of BWFE, moderate heterogeneity for the heritability estimate of BW, and considerable heterogeneities for the heritability estimates of BWG_g_ and BWG_gd_ (Table 3).

**Table 3:** Effect size and heterogeneity of the heritability estimates for economically important traits in ducks.

Trait*	Study	N	h^2^	SE	95 % CI	P-value	Q	P-value	I^2^
FI_g_	3	3	0.344	0.055	0.235-0.452	0.000	0.400	0.819	0.000
FI_gd_	6	9	0.288	0.059	0.173-0.404	0.000	126.484	0.000	93.675
RFI	5	6	0.266	0.034	0.199-0.332	0.000	2.963	0.706	0.000
DFR	2	3	0.540	0.040	0.461-0.618	0.000	2.857	0.240	30.002
NMD	2	3	0.568	0.025	0.519-0.618	0.000	0.070	0.965	0.000
MFI	2	3	0.624	0.025	0.574-0.673	0.000	0.203	0.904	0.000
MD	2	3	0.526	0.084	0.362-0.690	0.000	9.849	0.007	79.693
FCR	7	10	0.286	0.032	0.223-0.348	0.000	14.290	0.112	37.019
BW	23	69	0.396	0.013	0.371-0.421	0.000	126.508	0.000	46.248
BWFE	3	5	0.405	0.033	0.341-0.470	0.000	5.076	0.280	21.203
BWG_g_	4	5	0.154	0.058	0.041-0.268	0.008	15.934	0.003	74.897
BWG_gd_	5	9	0.321	0.095	0.134-0.508	0.001	85.019	0.000	90.590
EN	11	21	0.189	0.014	0.161-0.217	0.000	140.662	0.000	85.782
EW	7	14	0.350	0.021	0.309-0.392	0.000	121.557	0.000	89.305
ESS	2	3	0.119	0.006	0.108-0.131	0.000	0.479	0.787	0.000
EM_g_	2	3	0.303	0.052	0.202-0.404	0.000	0.095	0.953	0.000
EM_gd_	2	3	0.135	0.045	0.048-0.223	0.002	4.249	0.119	52.931
FER	2	3	0.271	0.085	0.106-0.437	0.001	24.904	0.000	91.969
HR	2	3	0.238	0.064	0.112-0.364	0.000	10.280	0.006	80.544
AFE	6	7	0.285	0.056	0.175-0.396	0.000	60.319	0.000	90.053
CW	3	3	0.512	0.038	0.437-0.587	0.000	0.366	0.833	0.000
LW	4	5	0.156	0.036	0.085-0.227	0.000	15.377	0.004	73.987
BMW	6	7	0.424	0.062	0.302-0.546	0.000	49.643	0.000	87.914
BMP	4	5	0.269	0.073	0.126-0.411	0.000	55.631	0.000	92.810
BMT	6	7	0.299	0.057	0.188-0.411	0.000	56.225	0.000	89.329
AFW	5	7	0.346	0.069	0.210-0.482	0.000	51.103	0.000	88.259
AFP	3	4	0.474	0.071	0.334-0.614	0.000	7.736	0.052	61.220
LMW	4	4	0.419	0.036	0.349-0.489	0.000	1.149	0.765	0.000
SFW	2	3	0.442	0.088	0.268-0.615	0.000	5.935	0.051	66.301
SFP	2	3	0.480	0.067	0.348-0.612	0.000	3.627	0.163	44.858

⁎ For traits, see Table 2. Study: Number of studies; N: Number of estimates

The heritability estimates for feeding traits were generally moderate to high and varied from 0.266 (for RFI) to 0.624 (for MFI), respectively. The standard errors of heritability estimates for feeding traits were generally small and the 95 % confidence intervals of these estimates were mostly narrow. Significant heritability estimates were also found for feeding traits (P < 0.05). The heterogeneity test of heritability estimates for growth traits, conducted by Q statistic, indicated that heritability estimates for FI_gd_ and MD had high Q values and significant heterogeneity (P < 0.10). However, the average heritability estimates for other feeding traits showed non-significant heterogeneity (P > 0.10). The I^2^ values demonstrated significant heterogeneity for the heritability estimates of FI_gd_ and MD, but only slight heterogeneity for the heritability estimates of the other feeding traits, which is consistent with the findings shown by the Q statistic (Table 3).

Results showed that heritability estimates for egg production and quality traits were generally low to moderate, ranging from 0.119 (for ESS) to 0.340 (for EW). The heritability estimates for egg production and quality traits exhibited small standard errors, and their 95 percent confidence intervals were largely narrow. Additionally, heritability estimates for egg production and quality traits were significant (P < 0.05). The results of the Q statistic heterogeneity test for egg production and quality traits showed that the heritability estimates for EN and EW had significant heterogeneity and high Q values (P < 0.10). Nonetheless, there was non-significant heterogeneity in the average heritability estimates for the other egg production and quality traits (P > 0.10). The I^2^ values revealed minor heterogeneity for the heritability estimates of ESS and EM_g_, moderate heterogeneity for the heritability estimate of EM_gd_, and significant heterogeneities for the heritability estimates of EN and EW, which were in line with the Q statistic results (Table 3).

The results of the meta-analysis showed that the heritability estimates for reproductive traits were moderate and were 0.271, 0.238 and 0.285 for FER, HR and AFE, respectively. The standard errors of heritability estimates for reproductive traits were generally small and the 95 % confidence intervals of these estimates were largely limited. Furthermore, estimates of heritability of reproductive traits were significant (P < 0.05). Heritability estimates for FER, HR, and AFE had high Q values and significant heterogeneity, according to the Q statistic's heterogeneity test of heritability estimates for reproduction traits (P < 0.10). The I^2^ values demonstrated considerable heterogeneity for the heritability estimates of all reproduction traits, which was consistent with the findings shown by the Q statistic.

Heritability estimates for carcass traits varied from low to high [between 0.156 (for LW) and 0.512 (for CW)]. All reproduction traits had low standard errors and mostly narrow 95 % confidence intervals for their heritability estimates. Furthermore, heritability estimates were significant for all reproductive traits (P < 0.05). The heritability estimates for CW, LMW, and SFP had low Q values and no significant heterogeneity, according to the heterogeneity test of the heritability estimates for reproductive traits conducted by Q statistics (P > 0.10). On the other hand, there were notable variations in the average heritability estimates for different carcass traits (P < 0.10). The I^2^ values revealed significant heterogeneities for the heritability estimates of other carcass traits and minor heterogeneity for the heritability estimates of CW and LMW, which were consistent with the results shown by the Q statistic (Table 3).

**Fig. 2:**
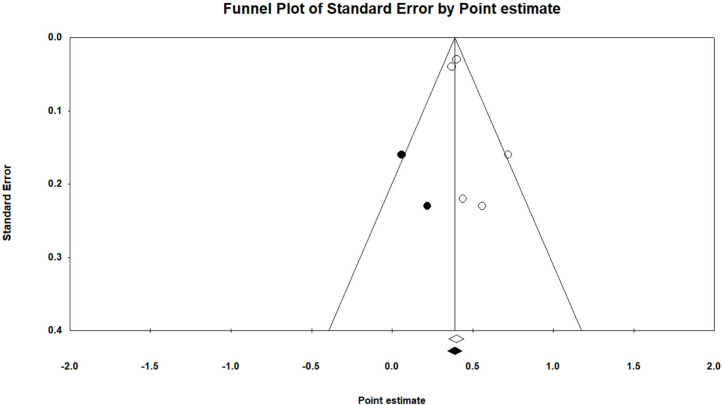
The funnel plot of the heritability estimate for BWFE. Solid dots represent the potentially missing studies that were found using the trim-and-fill method. When theoretically imputed studies are included in the meta-analysis, solid diamonds represent the mean values and confidence intervals, and open diamonds represent the mean values and confidence intervals for studies that are currently in the literature.

The forest plots of each study and the overall result for the heritability estimates of the investigated traits are displayed in Supplementary Figs 1 to 29. Furthermore, Fig 2 shows a funnel plot of the average heritability estimate for BWFE. Also, Supplementary Figs 30 to 41 show the funnel plots of the average heritability estimates for the other traits. Table 4 presents the findings from statistical tests to assess publication bias and the trim-and-fill technique to address funnel plot asymmetry in mean heritability estimates of traits that did not exhibit heterogeneity. For the mean heritability estimates of RFI and EM_g_, the Egger's test revealed a significant publication bias. For the heritability estimate of RFI to achieve funnel plot symmetry based on the trim-and-fill method, two missing studies were needed on the left side of the funnel plot. For the heritability estimates of MFI, BWFE, EM_gd_, and LMW, a non-significant (P > 0.10) publication bias was noted; however, in order to achieve funnel plot symmetry based on the trim-and-fill method, heritability estimates of these traits required two missing studies on the left side of the funnel plots. Following the inclusion of the imputed studies to correct the funnel plot asymmetry, the mean heritability estimates of RFI, MFI, BWFE, EM_gd_, and LMW were determined to be 0.256, 0.620, 0.389, 0.060, and 0.396, respectively (Table 4).

**Table 4:** Results of funnel plot asymmetry correction using trim-and-fill method and statistical tests to assess publication bias in mean heritability estimates of traits without heterogeneity.

Trait*	Egger's test p-value	Trim-and-fill method		
		Missing	Mean	95 % CI
FI_g_	0.392	0	0.344	0.235-0.452
RFI	0.020	2	0.256	0.191-0.322
DFR	0.810	0	0.540	0.461-0.618
NMD	0.440	0	0.568	0.519-0.618
MFI	0.249	2	0.620	0.574-0.666
FCR	0.527	0	0.286	0.223-0.348
BWFE	0.194	2	0.389	0.301-0.476
ESS	0.469	0	0.119	0.108-0.131
EM_g_	0.084	0	0.303	0.202-0.404
EM_gd_	0.370	2	0.060	-0.043-0.163
CW	0.893	0	0.512	0.437-0.587
LMW	0.254	2	0.396	0.335-0.458
SFP	0.618	0	0.480	0.348-0.612

⁎ For traits, see Table 2. Missing: Number of missing studies

### 3.3 Genetic correlation estimates

Table 5 displays the effect sizes and heterogeneity of genetic correlation estimates between various traits. Low genetic correlations of 0.230, 0.210, and 0.328 were found between BW-AFE, BW-HR, and BW-BWG (P < 0.05). Furthermore, the estimates of the genetic correlations between BW-EW, BW-BMW, BW-BMT, BW-LMW, BW-FI, and BW-EM were likewise moderate to high, with corresponding values of 0.629, 0.702, 0.484, 0.459, and 0.582, respectively (P < 0.05). There was no significant genetic correlation found between BW and other traits (P > 0.05). With the exception of the genetic correlation between BW-FI, which had a low Q value and non-significant heterogeneity (P > 0.10), the Q test heterogeneity tests of the genetic correlation estimates between BW and other traits revealed that the genetic correlation estimates between BW and other traits had large Q values and significant heterogeneity (P < 0.10). The I^2^ values showed minor heterogeneity for the genetic correlation between BW and FER, moderate heterogeneity for the genetic correlation between BW and FI, and significant heterogeneity between BW and other traits, all of which were consistent with the Q test results (Table 5).

**Table 5:** Effect sizes and heterogeneity of the genetic correlation estimates between different traits in ducks.

Trait 1*	Trait 2*	Study	N	r_g_	95 % CI	P-value	Q	P-value	I^2^
BW	EW	4	17	0.629	0.557-0.691	0.000	41.319	0.000	61.277
BW	EN	7	36	-0.048	-0.181-0.086	0.483	1321.154	0.000	97.351
BW	AFE	4	11	0.230	0.076-0.373	0.004	184.803	0.000	94.589
BW	FER	1	6	0.021	-0.064-0.107	0.624	10.302	0.067	51.464
BW	HR	1	6	0.210	0.074-0.338	0.003	22.989	0.000	78.251
BW	BMW	4	5	0.702	0.498-0.832	0.000	36.419	0.000	89.017
BW	BMT	5	10	0.484	0.256-0.661	0.000	62.521	0.000	85.605
BW	LMW	3	4	0.494	0.192-0.710	0.002	392.924	0.000	99.236
BW	LW	2	5	-0.078	-0.288-0.141	0.487	25.382	0.000	84.241
BW	FCR	5	8	0.055	-0.146-0.251	0.594	37.762	0.000	81.463
BW	FI	2	3	0.459	0.303-0.592	0.000	1.483	0.476	0.000
BW	RFI	4	6	0.070	-0.124-0.260	0.479	16.150	0.006	69.041
BW	BWG	6	24	0.328	0.191-0.451	0.000	70.345	0.000	67.304
BW	EM	2	6	0.582	0.313-0.765	0.000	1306.287	0.000	99.617
EW	EN	7	22	0.030	-0.105-0.165	0.663	87.258	0.000	75.933
EW	AFE	3	7	0.174	-0.044-0.376	0.118	26.984	0.000	77.765
EW	BWFE	2	3	0.580	0.269-0.781	0.001	25.117	0.000	92.037
EN	BWFE	2	3	0.273	-0.801-0.930	0.691	614.062	0.000	99.674
EN	AFE	5	12	-0.719	-0.853- -0.495	0.000	237.731	0.000	95.373
FI	FCR	5	7	0.318	0.150-0.468	0.000	23.162	0.001	74.096
FI	MFI	2	3	0.430	0.203-0.613	0.000	7.801	0.020	74.362
FI	DFR	2	3	0.164	0.072-0.253	0.001	0.136	0.934	0.000
FI	NM	2	3	-0.041	-0.203-0.122	0.621	3.416	0.181	41.451
FI	BWG	4	6	0.108	-0.060-0.269	0.208	12.181	0.032	58.951
FI	EM	2	3	0.501	0.285-0.669	0.000	2.969	0.227	32.644
FI	RFI	3	4	0.827	0.759-0.877	0.000	1.873	0.599	0.000
FCR	MFI	2	3	0.166	-0.007-0.329	0.060	5.704	0.058	64.934
FCR	RFI	3	4	0.565	0.486-0.634	0.000	1.747	0.627	0.000
FCR	DFR	2	3	0.126	0.021-0.228	0.018	0.936	0.626	0.000
FCR	NM	2	3	-0.037	-0.206-0.133	0.668	5.399	0.067	62.958
MFI	DFR	2	3	0.323	0.022-0.571	0.036	12.015	0.002	83.354
MFI	NM	2	3	-0.920	-0.952- -0.868	0.000	0.549	0.760	0.000
NM	DFR	2	3	-0.261	-0.630-0.205	0.270	25.094	0.000	92.030
BWG	EM	2	3	0.331	-0.279-0.751	0.285	12.766	0.002	84.334
BWG	RFI	5	6	-0.128	-0.235- -0.017	0.023	2.219	0.818	0.000
BWG	FCR	4	5	-0.301	-0.532- -0.029	0.031	28.480	0.000	85.955
EM	RFI	2	3	0.007	-0.059-0.072	0.844	1.598	0.450	0.000
BMW	BMP	3	3	0.664	0.495-0.784	0.000	5.313	0.070	62.356
BMW	LMW	4	4	0.542	0.305-0.716	0.000	19.352	0.000	84.497
BMW	AFW	3	3	0.235	-0.068-0.498	0.127	6.689	0.035	70.100
LMW	AFW	3	3	0.238	-0.036-0.479	0.088	5.241	0.073	61.841

⁎ r_g_: Genetic correlation; Study: Number of studies; N: Number of estimates

Based on the meta-analysis results, the genetic correlation between EW-BWFE was 0.580 (P < 0.05), but the genetic correlations between EW-EN and EW-AFE were not significant (P > 0.05). In contrast, the genetic correlation of EN-AFE was strong (-0.719, P < 0.05), but the genetic correlation between EN-BWFE was low (0.273) and not significant (P > 0.05). Moreover, the Q test and I^2^ statistic's heterogeneity tests of the genetic correlation estimates between EW-BWFE, EW-EN, EW-AFE, EN-AFE, and EN-BWFE revealed high heterogeneity (P < 0.10).

The results indicate that there was a high genetic correlation (0.827, P < 0.05) between FI-RFI, but low to moderate genetic correlations (P < 0.05) were found between FI-FCR, FI-MFI, FI-DFR, and FI-EM (0.318, 0.430, 0.164, and 0.501, respectively). It was found that there was no significant genetic correlation (P > 0.05) between FI-NM and FI-BWG. Genetic correlation estimates between FI-FCR, FI-MFI, and FI-BWG were subjected to heterogeneity tests using the Q test, which revealed significant heterogeneity (P < 0.10), but the genetic correlations between FI and other traits experienced non-significant heterogeneity (P > 0.10). The I^2^ values showed minor heterogeneity for the genetic correlations between FI-DFR, FI-EM, and FI-RFI, moderate heterogeneity for the genetic correlations between FI-NM and FI-BWG, and high heterogeneity between FI-FCR and FI-MFI, all of which were consistent with the Q test results (Table 5).

The findings demonstrated that the FCR-RFI, FCR-DFR, MFI-DFR, and MFI-NM had genetic correlations of 0.565, 0.126, 0.323, and -0.920, respectively (P < 0.05). Additionally, using the Q test and I2 statistic, the heterogeneity tests of genetic correlation estimates between FCR-RFI, FCR-DFR, and MFI-NM revealed minor heterogeneity (P > 0.10), but they also revealed high heterogeneity between the genetic correlations of FCR-MFI, FCR-NM, MFI-DFR, and NM-DFR (P < 0.10).

The genetic correlation estimates between BWG-RFI and BWG-FCR were -0.128 and -0.301, respectively (P < 0.05), but the genetic correlations between BWG-EM and EM-RFI were low and not significant (P > 0.05). The genetic correlations between BWG-EM and BWG-FCR had high Q values and significant heterogeneity (P < 0.10) according to heterogeneity tests of genetic correlation estimates conducted by the Q test, but the genetic correlations between BWG-RFI and EM-RFI experienced non-significant heterogeneity (P > 0.10). The I^2^ values showed minimal heterogeneity for the genetic correlations between BWG-RFI and EM-RFI, but high heterogeneity for the genetic correlations between BWG-EM and BWG-FCR, which was in line with the Q test results (Table 5).

The findings revealed that BMW-BMP and BMW-LMW had genetic correlations of 0.664 and 0.542, respectively (P < 0.05), but the genetic correlations between BMW-AFW and LMW-AFW were low and not significant (P > 0.05). The 95 % confidence interval contained zero for genetic correlation estimates that were not statistically significant. These estimates cannot, therefore, be statistically distinguished from zero. There was a considerable amount of heterogeneity for the genetic correlations between BMW-BMP, BMW-LMW, BMW-AFW, and LMW-AFW, according to the heterogeneity tests of the genetic correlation estimates carried out by the Q test and I^2^ statistic (Table 5; P < 0.10).

**Fig. 3:**
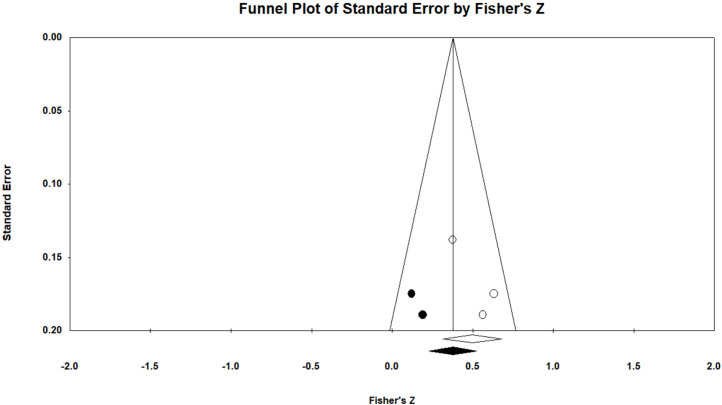
The funnel plot of the genetic correlation estimate between BW-FI. Details are shown in Fig. 1.

The forest plots of the estimated genetic correlations between traits are shown in Supplementary Figs 42 to 83. Furthermore, Fig. 3 shows a funnel plot of the average genetic correlation estimate between BW-FI. Moreover, the funnel plots of the estimated genetic correlations between the other traits are shown in Supplementary Figs 84 to 92. Table 6 displays the findings from the statistical tests used to investigate publication bias as well as the trim-and-fill technique used to correct for the asymmetry of the funnel plot in the genetic correlation estimate between traits that did not exhibit heterogeneity. According to Egger's test, the genetic correlation estimates between FI-NM and FCR-DFR were found to have significant publication bias (P < 0.10). However, no publication bias could be identified in the test results for other genetic connections. In order to obtain funnel plot symmetry based on the trim-and-fill method, genetic correlation between BW-FI required two missing studies on the left side of the funnel plot. Furthermore, for genetic correlations between FI-NM and FCR-DFR, two missing studies were needed on the right side of the funnel plot. Additionally, to obtain funnel plot symmetry based on the trim-and-fill method, one missing study was required on the left side of the funnel plot for genetic correlations between FI-RFI, FCR-RFI, and EM-RFI, and one missing study was required on the right side of the funnel plot for genetic correlations between MFI-NM and BWG-RFI. The mean genetic correlation estimates between BW-FI, FI-NM, FCR-DFR, FI-RFI, FCR-RFI, EM-RFI, MFI-NM, and BWG-RFI were 0.360, 0.050, 0.150, 0.821, 0.565, 0.005, -0.919, and -0.126, respectively, after the funnel plot asymmetry was corrected by including the imputed studies (Table 6).

**Table 6:** Assessment of publication bias by Egger's test and the implementation of the trim-and-fill method to correct the funnel plot asymmetry of genetic correlation between traits that did not show heterogeneity.

Trait 1*	Trait 2*	Egger's test p-value	Trim-and-fill method		
			Missing	Mean	95 % CI
BW	FI	0.294	2	0.360	0.187-0.511
FI	DFR	0.703	0	0.164	0.072-0.253
FI	NM	0.094	2	0.050	-0.106-0.203
FI	EM	0.853	0	0.501	0.285-0.669
FI	RFI	0.755	1	0.821	0.754-0.871
FCR	RFI	0.935	1	0.565	0.486-0.634
FCR	DFR	0.070	2	0.150	0.055-0.242
MFI	NM	0.835	1	-0.919	-0.951- -0.867
BWG	RFI	0.924	1	-0.126	-0.233- -0.016
EM	RFI	0.556	1	0.005	-0.060-0.070

⁎ For traits, see Table 2. Missing: Number of missing studies.

## 4 Discussion

Since genetic information is passed from parents to offspring, genetic improvement of ducks is a practical means of increasing their productivity. Therefore, in order to boost duck productivity, certain selection schemes are needed. Economically important characteristics like growth, carcass, reproduction, feeding, and egg production and quality are among the selection criteria for ducks. For the purpose of creating breeding programs, forecasting selection response, and elucidating genetic effects, the availability of precise and trustworthy genetic parameters for quantitative traits is crucial. Evaluating the additive genetic variation of economically significant traits and estimating their genetic associations are essential steps in developing effective genetic evaluation and improvement plans for ducks. Clearly, in order to improve duck breeding strategies relatively quickly and significantly, economically important traits need to be sufficiently heritable.

The weighted coefficient of variation for the majority of the studied traits ranged from low to moderate. With little variation around the weighted mean of the trait across studies, this low to moderate weighted coefficient of variation indicates that these traits show limited phenotypic variation. As a result, the weighted average estimate of HR, which has the lowest weighted coefficient of variation (0.82 %), is considered to be more accurate. AFW, MD, and EN were found to have the greatest weighted coefficient of variation when compared to other duck traits, indicating a higher degree of phenotypic variation in these characteristics.

The heritability estimates of the majority of traits under study generally show good precision, as evidenced by their low standard error and 95 % confidence interval. The results of this meta-analysis showed moderate to high heritability estimates for feeding traits (from 0.266 to 0.624), low to high heritability estimates for growth (from 0.154 to 0.405) and carcass traits (from 0.156-0.512), low to moderate heritability estimates for egg production and quality traits (from 0.119-0.340), and moderate heritability estimates for reproduction traits (from 0.238 to 0.285). Some groups of traits had low heritability estimates, indicating a greater influence from non-genetic factors. However, the majority of the traits under study have moderate to high average heritability estimates, indicating moderate to high additive genetic influences on the traits' expression. Furthermore, the presence of available additive genetic variation for these traits, which can be utilized for genetic selection strategies, was indicated by moderate to high average heritability estimates. Heritability is one of the primary factors that determines the rate of genetic advancement, along with genetic variation, selection intensity, and generation interval. It is possible that the actual parameters in a meta-analysis will differ from study to study because it aggregated published genetic parameter estimates from various studies. Diverse factors, including sample size, breeds employed, analysis models and techniques, and variation resulting from trait measurement, may contribute to the disparities in genetic parameter estimates reported in various studies (Ghavi Hossein-Zadeh, 2024).

Higher efficacy is associated with lower food intake, as evidenced by the strong and positive genetic correlation between FI-RFI and the moderately positive genetic correlation between FCR-RFI. However, this study found little genetic correlation between BW-RFI and BWG-RFI. This is a very interesting result because selection based on RFI would reduce FI without changing BW's genetic makeup (Basso et al., 2012). Thus, it could be deduced that choosing ducks with low RFI levels would help select for high feed efficiency while having minimal impact on the rise in BW. Feeding the ducks is the main farming expense, as it is for all livestock, particularly considering their lengthy lifespan. Therefore, feed efficiency is a critical economic feature for breeders. FCR was once thought to be the primary criterion for feed efficiency in ducks; however, because the trait's component traits may not be linear, improving this trait genetically is difficult and may result in unfavorable outcomes, such as altered BW and egg production in laying hens (Bordas et al., 1992). Thus, in an effort to increase feed efficiency in ducks, the idea of RFI was explored. To calculate feed efficiency independently of feed requirements for production – which may include weight gain, carcass yield or fat deposition, as well as feed requirements for maintenance – a linear combination of characteristics called RFI is used (Drouilhet et al., 2014). RFI is, by definition, a portion of total food intake that is phenotypically independent of energy needs for production and maintenance. However, depending on the species and animal studies, the genetic correlations between RFI and its constituent parts vary. The application of this selection criterion in birds, such as laying hens, and mammals has amply demonstrated its efficacy (Bordas et al., 1992), and a number of investigations have been conducted to assess any potential genetic or physiological effects (Basso et al., 2012; Tixier-Boichard et al., 2002).

A higher NM does not necessarily translate into a higher MFI, as evidenced by the strong and negative genetic correlation between MFI-NM. An increase in meal frequency, if the animal has reached satiety, only serves to highlight the animal's variable feeding behavior rather than an urge to consume more feed (Chapuis et al., 2022). Ducks with a high DFR may sate quickly and not feel the need to begin a new feeding sequence, according to the negative genetic correlation found between NM-DFR (Chapuis et al., 2022). Ducks' feeding behavior, including MFI, NM, and DFR, is a crucial characteristic. On the one hand, feeding behavior can reveal information about ducks' dietary preferences and physiological state. The meta-analysis's findings showed that MFI and NM had a positive genetic relationship with FCR in ducks. In current broiler lines, there is also a strong correlation between feeding behavior and FCR (Howie and Tolkamp, 2009). There are also notable differences in feeding behavior between the Pekin duck groups with low and high RFI levels (Drouilhet et al., 2016). While feeding behavior in ducks offers a great deal of potential for realizing indirect selection for feed efficiency, gathering data on feeding behavior in previous breeding programs has proven to be a laborious and complicated process. Researchers can now precisely and automatically record an individual's feeding behavior thanks to the widespread use of electronic feeders. This allows them to learn more about the genetic relationships between feed efficiency, body weight, and feeding behavior (Li et al., 2020).

Given the positive correlation between BW and HR, it is possible that duckling hatchability is influenced by growth during the embryonic stage (Brun and Larzul, 2003). However, because of the weak genetic correlation between BW and FER, selection for BW in duck breeding programs could not affect the FER. The BW-EW genetic correlation was found to be moderately to highly positive, suggesting that larger ducks typically lay larger eggs. Conversely, weak (near zero) genetic associations were found between BW-EN and EN-EW, suggesting that ducks could be selected to increase EN without negatively affecting EW and maintaining BW. Enhancing egg-laying traits is crucial because they have a significant impact on duckling production (Hu et al., 2004). A strong and negative genetic correlation was found between EN-AFE, while a positive and low genetic correlation was found between BW-AFE, according to the meta-analysis's findings. That meant larger ducks with lower EN would come from higher AFE. Due to moderate to high genetic correlations between BW-BMW, BW-LMW, BW-BMT, and BMW-LMW, the results also showed that larger ducks had greater BMW, LMW, and BMT. The joint genetic and physiological mechanisms directing traits are demonstrated by the positive (and particularly moderate to high) genetic correlations between traits. The 95 percent confidence interval included zero for the genetic correlation estimates that were not significant, and this should be carefully interpreted. The lack of a genetic correlation implies that the selection of one trait cannot significantly alter another trait, and vice versa.

In the current meta-analysis, the majority of genetic parameter estimates showed moderate to significant heterogeneity. Because of this heterogeneity, using a random-effects model to take study differences into account was justified. The implementation of a random-effects model to account for variations across studies was justified by this heterogeneity. The differentiation between estimates is generally influenced by a number of factors, including animal breed, genetic variation within the population, management and environmental conditions, and parameter estimation methods/models. These factors are considered to be the potential causes of heterogeneity in the meta-analysis. Furthermore, in this meta-analysis, asymmetry was shown in a few funnel plots. Asymmetrical funnel plots may be a sign of publication bias or the result of treatment effects in small, poorly conducted studies being overstated. Egger and Smith (2001) reported that the following categories typically comprise the possible causes of asymmetry in funnel plots: 1. Biases in selection, 2. Real heterogeneity (differences in effect size based on study size), 3. Inconsistent data, 4. Artefact (inadequate effect measure selection leading to heterogeneity), and 5. Chance.

## 5 Conclusions

This meta-analysis provides the first comprehensive genetic assessment of economically important traits in ducks, covering growth, feeding, egg production, reproduction, and carcass traits. Key results showed moderate to high heritability estimates for growth and nutritional traits, suggesting potential for genetic progress in these areas. In particular, selecting ducks with low RFI promises to improve feed efficiency without significant impact on BW. Across egg production, reproductive and carcass traits, heritability estimates vary widely, highlighting both genetic potential and trait-specific selection challenges. Strong genetic correlations, particularly between traits such as FI and FCR, highlight opportunities for simultaneous improvement in these economically important traits. Ultimately, these results highlight important genetic relationships between growth, feeding, reproductive and egg quality traits that can serve as a basis for balanced selection strategies aimed at improving overall productivity and efficiency in duck populations.

## Compliance with ethical standards

### Ethical approval

This article does not contain any studies with human participants performed by any of the authors.

## Funding

None.

## CRediT authorship contribution statement

**Navid Ghavi Hossein-Zadeh:** Writing – review & editing, Writing – original draft, Visualization, Validation, Supervision, Project administration, Methodology, Investigation, Formal analysis, Data curation, Conceptualization.

## Declaration of competing interest

The authors declare that they have no known competing financial interests or personal relationships that could have appeared to influence the work reported in this paper.

## Appendix Supplementary materials

Image, application 1
