# Supplementary material for: A meta-analysis of genetic estimates for economically important traits in ducks
Source: Vet Anim Sci. 2024 Nov 1;26:100405. doi: 10.1016/j.vas.2024.100405 (PMC11576399; doi:10.1016/j.vas.2024.100405)
Supplement: Supplementary file 1 [file mmc1.pdf]

Study name	Point estimate	Standard error	Variance	Lower limit	Upper limit	Z-Value	p-Value
Brun and Lazrul (2003)	0.470	0.040	0.002	0.392	0.548	11.750	0.000
Cheng et al. (1995)	0.188	0.004	0.000	0.180	0.196	47.000	0.000
Damayanti et al. (2019)- Data set 1	0.590	0.230	0.053	0.139	1.041	2.565	0.010
Damayanti et al. (2019)- Data set 2	0.840	0.240	0.058	0.370	1.310	3.500	0.000
Hu et al. (2004)	0.200	0.030	0.001	0.141	0.259	6.667	0.000
Lin et al. (2016)	0.128	0.076	0.006	-0.021	0.277	1.684	0.092
Tai et al. (1989)	0.180	0.130	0.017	-0.075	0.435	1.385	0.166
	0.285	0.056	0.003	0.175	0.396	5.083	0.000

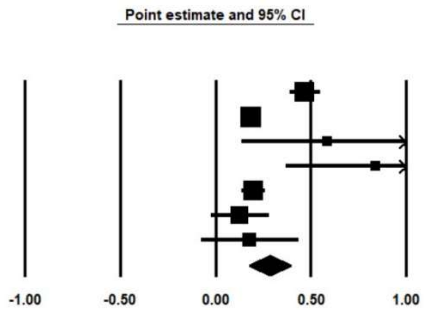

Supplementary Fig. 1. The forest plot of individual studies and the overall outcome for heritability estimate of AFE. The mean effect size, calculated according to a random-effects model, is indicated by the diamond at the bottom of each plot. The size of the squares illustrates the weight of each study relative to the mean effect size. Smaller squares represent less weight. The horizontal bars represent the 95% confidence intervals for the study.

Study name	Point estimate	Standard error	Variance	Lower limit	Upper limit	Z-Value	p-Value
Cai et al. (2023)	0.560	0.080	0.006	0.403	0.717	7.000	0.000
Xu et al. (2011)	0.320	0.060	0.004	0.202	0.438	5.305	0.000
Zhang et al. (2022)- Data set 1	0.560	0.120	0.014	0.325	0.795	4.667	0.000
Zhang et al. (2022)- Data set 2	0.520	0.110	0.012	0.304	0.736	4.727	0.000
	0.474	0.071	0.005	0.334	0.614	6.650	0.000

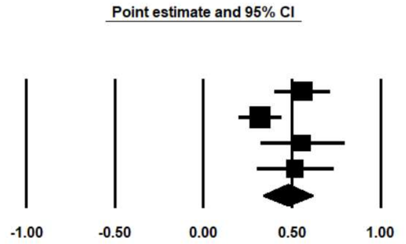

Supplementary Fig. 2. The forest plot of individual studies and the overall outcome for heritability estimate of AFP. Details are provided in Supplementary Fig. 1.

Study name	Point estimate	Standard error	Variance	Lower limit	Upper limit	Z-Value	p-Value
Cai et al. (2023)	0.630	0.080	0.006	0.473	0.787	7.875	0.000
Marie-Etancelin et al. (2011)- Data set 1	0.250	0.040	0.002	0.172	0.328	6.250	0.000
Marie-Etancelin et al. (2011)- Data set 2	0.090	0.040	0.002	0.012	0.168	2.250	0.024
Mucha et al. (2014)	0.470	0.160	0.026	0.156	0.784	2.938	0.003
Xu et al. (2011)	0.230	0.048	0.002	0.136	0.324	4.799	0.000
Zhang et al. (2022)- Data set 1	0.520	0.090	0.008	0.344	0.696	5.778	0.000
Zhang et al. (2022)- Data set 2	0.360	0.090	0.008	0.184	0.536	4.000	0.000
	0.346	0.069	0.005	0.210	0.482	4.981	0.000

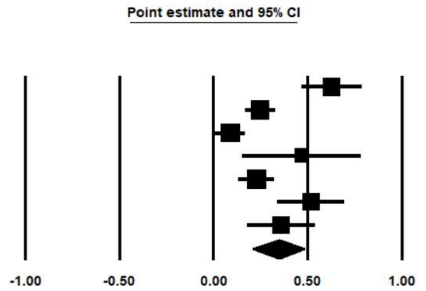

Supplementary Fig. 3. The forest plot of individual studies and the overall outcome for heritability estimate of AFW. Details are provided in Supplementary Fig. 1.

Study name	Point estimate	Standard error	Variance	Lower limit	Upper limit	Z-Value	p-Value
Cai et al. (2023)	0.380	0.070	0.005	0.243	0.517	5.429	0.000
Xu et al. (2011)	0.470	0.042	0.002	0.387	0.553	11.135	0.000
Xu et al. (2018)	0.160	0.016	0.000	0.128	0.192	9.912	0.000
Zhang et al. (2022)- Data set 1	0.130	0.050	0.003	0.032	0.228	2.600	0.009
Zhang et al. (2022)- Data set 2	0.210	0.080	0.006	0.053	0.367	2.625	0.009
	0.202	0.014	0.000	0.174	0.229	14.482	0.000

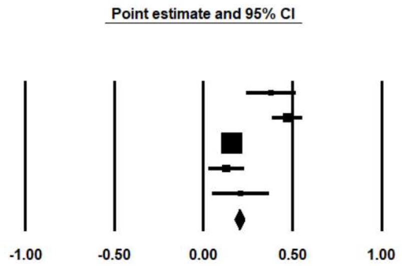

Supplementary Fig. 4. The forest plot of individual studies and the overall outcome for heritability estimate of BMP. Details are provided in Supplementary Fig. 1.

Study name	Point estimate	Standard error	Variance	Lower limit	Upper limit	Z-Value	p-Value
	Statistics for each study						
Cai et al. (2024)	0.330	0.070	0.005	0.193	0.467	4.714	0.000
Duggan et al. (2017)- Data set 1	0.150	0.074	0.005	0.005	0.295	2.027	0.043
Duggan et al. (2017)- Data set 2	0.295	0.046	0.002	0.205	0.385	6.413	0.000
Szwaczkowski et al. (2010)	0.247	0.041	0.002	0.167	0.327	6.024	0.000
Wähner et al. (2017)	0.550	0.076	0.006	0.401	0.699	7.258	0.000
Xu et al. (2011)	0.450	0.063	0.004	0.326	0.574	7.132	0.000
Xu et al. (2018)	0.120	0.024	0.001	0.073	0.167	5.022	0.000
	0.299	0.057	0.003	0.188	0.411	5.246	0.000

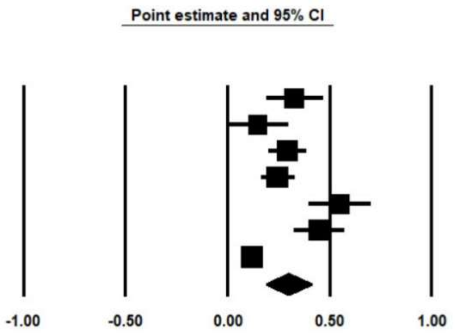

Supplementary Fig. 5. The forest plot of individual studies and the overall outcome for heritability estimate of BMT. Details are provided in Supplementary Fig. 1.

Study name	Point estimate	Standard error	Variance	Lower limit	Upper limit	Z-Value	p-Value
	Statistics for each study						
Cai et al. (2023)	0.440	0.070	0.005	0.303	0.577	6.286	0.000
Mucha et al. (2014)	0.690	0.170	0.029	0.357	1.023	4.059	0.000
Wähner et al. (2017)	0.580	0.061	0.004	0.460	0.700	9.482	0.000
Xu et al. (2011)	0.500	0.051	0.003	0.400	0.600	9.818	0.000
Xu et al. (2018)	0.260	0.019	0.000	0.222	0.298	13.480	0.000
Zhang et al. (2022)- Data set 1	0.390	0.080	0.006	0.233	0.547	4.875	0.000
Zhang et al. (2022)- Data set 2	0.250	0.070	0.005	0.113	0.387	3.571	0.000
	0.424	0.062	0.004	0.302	0.546	6.799	0.000

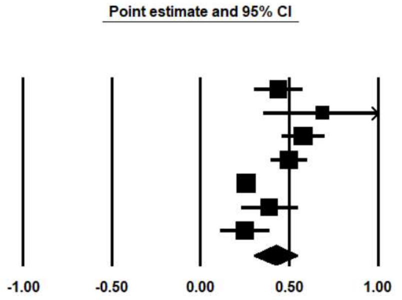

Supplementary Fig. 6. The forest plot of individual studies and the overall outcome for heritability estimate of BMW. Details are provided in Supplementary Fig. 1.

Study name	Point estimate	Standard error	Variance	Lower limit	Upper limit	Z-Value	p-Value
	Statistics for each study						
Damayanti et al. (2019)- Data set 1	0.560	0.230	0.053	0.109	1.011	2.435	0.015
Damayanti et al. (2019)- Data set 2	0.440	0.220	0.048	0.009	0.871	2.000	0.046
El-Deghadi et al. (2022)- Data set 1	0.370	0.040	0.002	0.292	0.448	9.250	0.000
El-Deghadi et al. (2022)- Data set 2	0.400	0.030	0.001	0.341	0.459	13.333	0.000
Qiangqiang et al. (2022)	0.720	0.160	0.026	0.406	1.034	4.500	0.000
	0.405	0.033	0.001	0.341	0.470	12.289	0.000

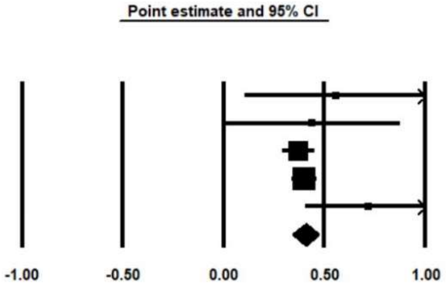

Supplementary Fig. 7. The forest plot of individual studies and the overall outcome for heritability estimate of BWFE. Details are provided in Supplementary Fig. 1.

Study name	Point estimate	Standard error	Variance	Lower limit	Upper limit	Z-Value	p-Value
Drouilhet et al. (2014)	0.390	0.220	0.048	-0.041	0.821	1.773	0.076
Li et al. (2020)	0.370	0.095	0.009	0.183	0.557	3.887	0.000
Marie-Etancelin et al. (2011)- Data set 1	0.060	0.030	0.001	0.001	0.119	2.000	0.046
Marie-Etancelin et al. (2011)- Data set 2	0.040	0.030	0.001	-0.019	0.099	1.333	0.182
Wähner et al. (2017)	0.390	0.192	0.037	0.014	0.766	2.032	0.042
	0.154	0.058	0.003	0.041	0.268	2.667	0.008

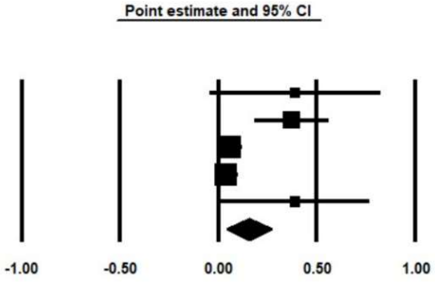

Supplementary Fig. 8. The forest plot of individual studies and the overall outcome for heritability estimate of BWG_g. Details are provided in Supplementary Fig. 1.

Study name	Point estimate	Standard error	Variance	Lower limit	Upper limit	Z-Value	p-Value
Basso et al. (2012)	0.090	0.130	0.017	-0.165	0.345	0.692	0.489
Cai et al. (2024)- Data set 1	0.570	0.160	0.026	0.256	0.884	3.563	0.000
Cai et al. (2024)- Data set 2	0.560	0.140	0.020	0.286	0.834	4.000	0.000
Cai et al. (2024)- Data set 3	0.530	0.080	0.006	0.373	0.687	6.625	0.000
Cai et al. (2024)- Data set 4	0.210	0.120	0.014	-0.025	0.445	1.750	0.080
Cai et al. (2024)- Data set 5	0.240	0.150	0.023	-0.054	0.534	1.600	0.110
Li et al. (2020)	0.360	0.080	0.006	0.203	0.517	4.496	0.000
Zeng et al. (2018)- Data set 1	0.030	0.010	0.000	0.010	0.050	3.000	0.003
Zhang et al. (2017)	0.380	0.200	0.040	-0.012	0.772	1.900	0.057
	0.321	0.095	0.009	0.134	0.508	3.369	0.001

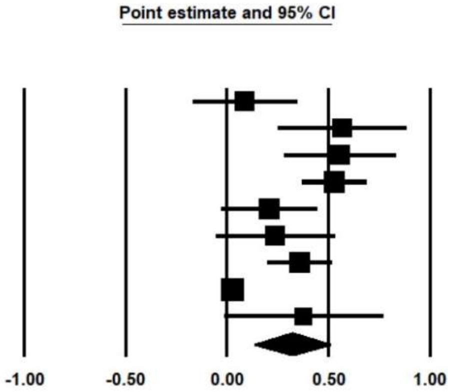

Supplementary Fig. 9. The forest plot of individual studies and the overall outcome for heritability estimate of BWG_{gd}. Details are provided in Supplementary Fig. 1.

Study name	Point estimate	Standard error	Variance	Lower limit	Upper limit	Z-Value	p-Value
Cai et al. (2023)	0.540	0.080	0.006	0.383	0.697	6.750	0.000
Wähner et al. (2017)	0.480	0.068	0.005	0.347	0.613	7.084	0.000
Xu et al. (2011)	0.520	0.056	0.003	0.409	0.631	9.218	0.000
	0.512	0.038	0.001	0.437	0.587	13.430	0.000

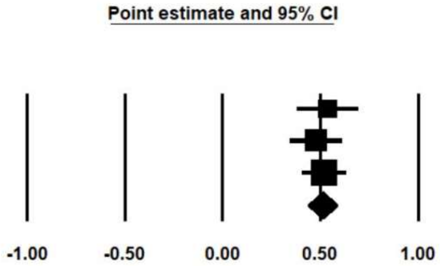

Supplementary Fig. 10. The forest plot of individual studies and the overall outcome for heritability estimate of CW. Details are provided in Supplementary Fig. 1.

Study name	Point estimate	Standard error	Variance	Lower limit	Upper limit	Z-Value	p-Value
Chapuis et al. (2022)- Data set 1	0.610	0.070	0.005	0.473	0.747	8.714	0.000
Chapuis et al. (2022)- Data set 2	0.390	0.110	0.012	0.174	0.606	3.545	0.000
Li et al. (2020)	0.540	0.028	0.001	0.485	0.595	19.321	0.000
	0.540	0.040	0.002	0.461	0.618	13.410	0.000

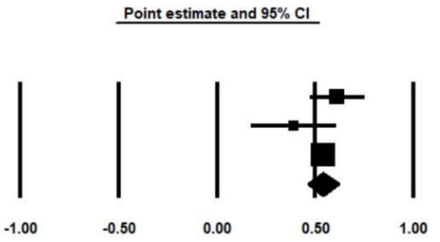

Supplementary Fig. 11. The forest plot of individual studies and the overall outcome for heritability estimate of DFR. Details are provided in Supplementary Fig. 1.

Study name	Point estimate	Standard error	Variance	Lower limit	Upper limit	Z-Value	p-Value
Boonlert et al. (2005)	0.385	0.270	0.073	-0.144	0.914	1.426	0.154
El-Deghadi et al. (2022)- Data set 1	0.300	0.080	0.006	0.143	0.457	3.750	0.000
El-Deghadi et al. (2022)- Data set 2	0.300	0.070	0.005	0.163	0.437	4.286	0.000
	0.303	0.052	0.003	0.202	0.404	5.862	0.000

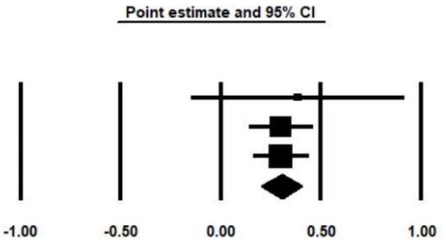

Supplementary Fig. 12. The forest plot of individual studies and the overall outcome for heritability estimate of EM_g. Details are provided in Supplementary Fig. 1.

Study name	Point estimate	Standard error	Variance	Lower limit	Upper limit	Z-Value	p-Value
Basso et al. (2012)	0.060	0.050	0.003	-0.038	0.158	1.200	0.230
Zeng et al. (2018)- Data set 1	0.220	0.060	0.004	0.102	0.338	3.667	0.000
Zeng et al. (2018)- Data set 2	0.140	0.050	0.003	0.042	0.238	2.800	0.005
	0.135	0.045	0.002	0.048	0.223	3.029	0.002

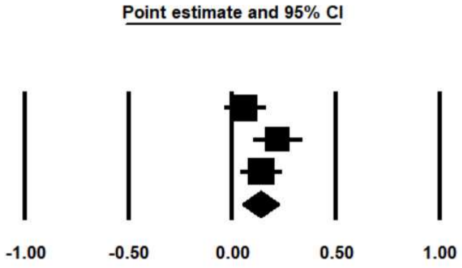

Supplementary Fig. 13. The forest plot of individual studies and the overall outcome for heritability estimate of EM_{gd}. Details are provided in Supplementary Fig. 1.

Study name	Point estimate	Standard error	Variance	Lower limit	Upper limit	Z-Value	p-Value
	Statistics for each study						
Basso et al. (2012)	0.200	0.110	0.012	-0.016	0.416	1.818	0.069
Boonlert et al. (2005)	0.099	0.030	0.001	0.040	0.158	3.300	0.001
Brun and Lazrul (2003)	0.240	0.030	0.001	0.181	0.299	8.000	0.000
Cheng et al. (1995)- Data set 1	0.146	0.004	0.000	0.138	0.154	36.500	0.000
Cheng et al. (1995)- Data set 2	0.111	0.003	0.000	0.105	0.117	37.000	0.000
Damayanti et al. (2019)- Data set 1	0.620	0.230	0.053	0.169	1.071	2.696	0.007
Damayanti et al. (2019)- Data set 2	0.460	0.220	0.048	0.029	0.891	2.091	0.037
El-Deghadi et al. (2022)- Data set 1	0.240	0.090	0.008	0.064	0.416	2.667	0.008
El-Deghadi et al. (2022)- Data set 2	0.290	0.090	0.008	0.114	0.466	3.222	0.001
Hu et al. (2004)- Data set 1	0.230	0.030	0.001	0.171	0.289	7.667	0.000
Hu et al. (2004)- Data set 2	0.270	0.030	0.001	0.211	0.329	9.000	0.000
Hu et al. (2004)- Data set 3	0.200	0.030	0.001	0.141	0.259	6.667	0.000
Hu et al. (2004)- Data set 4	0.220	0.030	0.001	0.161	0.279	7.333	0.000
Lin et al. (2016)- Data set 1	0.425	0.139	0.019	0.153	0.697	3.058	0.002
Lin et al. (2016)- Data set 2	0.428	0.152	0.023	0.130	0.726	2.816	0.005
Lin et al. (2016)- Data set 3	0.376	0.147	0.022	0.088	0.664	2.558	0.011
Liu et al. (2021)	0.100	0.187	0.035	-0.266	0.466	0.536	0.592
Qiangqiang et al. (2022)	0.150	0.170	0.029	-0.183	0.483	0.882	0.378
Tai et al. (1989)- Data set 1	0.140	0.120	0.014	-0.095	0.375	1.167	0.243
Tai et al. (1989)- Data set 2	0.120	0.120	0.014	-0.115	0.355	1.000	0.317
Tai et al. (1989)- Data set 3	0.020	0.110	0.012	-0.196	0.236	0.182	0.856
	0.189	0.014	0.000	0.161	0.217	13.277	0.000

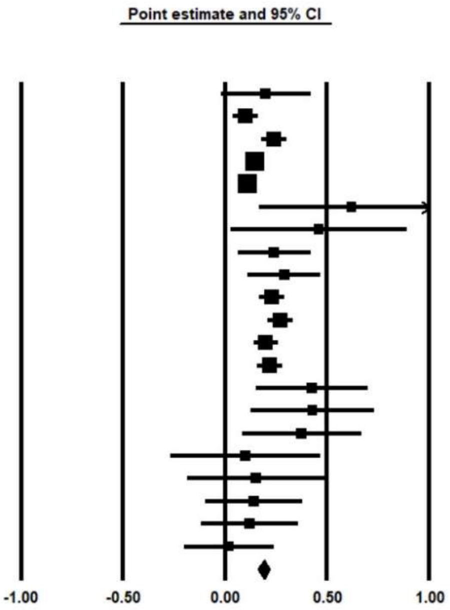

Supplementary Fig. 14. The forest plot of individual studies and the overall outcome for heritability estimate of EN. Details are provided in Supplementary Fig. 1.

Study name	Point estimate	Standard error	Variance	Lower limit	Upper limit	Z-Value	p-Value
	Statistics for each study						
Cheng et al. (1995)- Data set 1	0.094	0.037	0.001	0.021	0.167	2.519	0.012
Cheng et al. (1995)- Data set 2	0.120	0.006	0.000	0.108	0.132	20.000	0.000
Tai et al. (1989)	0.110	0.120	0.014	-0.125	0.345	0.917	0.359
	0.119	0.006	0.000	0.108	0.131	20.167	0.000

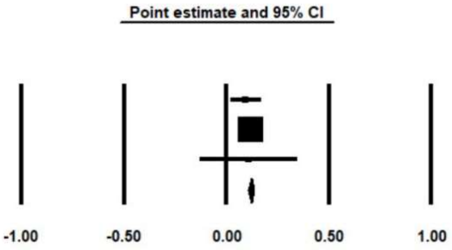

Supplementary Fig. 15. The forest plot of individual studies and the overall outcome for heritability estimate of ESS. Details are provided in Supplementary Fig. 1.

Study name	Point estimate	Standard error	Variance	Lower limit	Upper limit	Z-Value	p-Value
Boonlert et al. (2005)	0.332	0.190	0.036	-0.040	0.704	1.747	0.081
Cheng et al. (1995)- Data set 1	0.304	0.004	0.000	0.296	0.312	76.000	0.000
Cheng et al. (1995)- Data set 2	0.353	0.003	0.000	0.347	0.359	117.667	0.000
El-Deghadi et al. (2022)- Data set 1	0.210	0.080	0.006	0.053	0.367	2.625	0.009
El-Deghadi et al. (2022)- Data set 2	0.240	0.100	0.010	0.044	0.436	2.400	0.016
Lin et al. (2016)- Data set 1	0.426	0.108	0.012	0.214	0.638	3.944	0.000
Lin et al. (2016)- Data set 2	0.468	0.131	0.017	0.211	0.725	3.573	0.000
Lin et al. (2016)- Data set 3	0.507	0.128	0.016	0.256	0.758	3.961	0.000
Lin et al. (2016)- Data set 4	0.614	0.148	0.022	0.324	0.904	4.149	0.000
Lin et al. (2016)- Data set 5	0.652	0.093	0.009	0.470	0.834	7.011	0.000
Liu et al. (2021a)	0.710	0.220	0.048	0.278	1.142	3.224	0.001
Qiangqiang et al. (2022)	0.290	0.190	0.036	-0.082	0.662	1.526	0.127
Tai et al. (1989)- Data set 1	0.320	0.150	0.023	0.026	0.614	2.133	0.033
Tai et al. (1989)- Data set 2	0.240	0.130	0.017	-0.015	0.495	1.846	0.065
	0.350	0.021	0.000	0.309	0.392	16.441	0.000

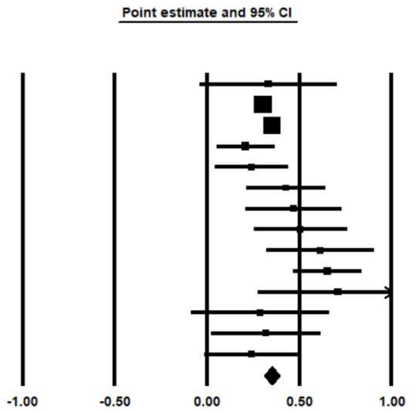

Supplementary Fig. 16. The forest plot of individual studies and the overall outcome for heritability estimate of EW. Details are provided in Supplementary Fig. 1.

Study name	Point estimate	Standard error	Variance	Lower limit	Upper limit	Z-Value	p-Value
Chapuis et al. (2022)- Data set 1	0.480	0.070	0.005	0.343	0.617	6.857	0.000
Chapuis et al. (2022)- Data set 2	0.250	0.090	0.008	0.074	0.426	2.778	0.005
Drouilhet et al. (2014)	0.410	0.180	0.032	0.057	0.763	2.278	0.023
Duggan et al. (2017)- Data set 1	0.272	0.096	0.009	0.084	0.460	2.833	0.005
Duggan et al. (2017)- Data set 2	0.294	0.048	0.002	0.200	0.388	6.125	0.000
Li et al. (2020)	0.290	0.061	0.004	0.170	0.410	4.736	0.000
Liu et al. (2021)	0.230	0.252	0.064	-0.264	0.724	0.912	0.362
Zeng et al. (2018)- Data set 1	0.190	0.050	0.003	0.092	0.288	3.800	0.000
Zeng et al. (2018)- Data set 2	0.190	0.070	0.005	0.053	0.327	2.714	0.007
Zhang et al. (2017)	0.380	0.150	0.023	0.086	0.674	2.533	0.011
	0.286	0.032	0.001	0.223	0.348	8.890	0.000

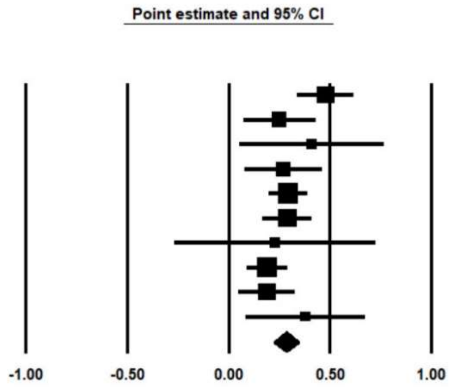

Supplementary Fig. 17. The forest plot of individual studies and the overall outcome for heritability estimate of FCR. Details are provided in Supplementary Fig. 1.

Study name — **Statistics for each study**

Study name	Point estimate	Standard error	Variance	Lower limit	Upper limit	Z-Value	p-Value
Li et al. (2020)	0.340	0.066	0.004	0.210	0.470	5.144	0.000
Liu et al. (2021)	0.510	0.272	0.074	-0.023	1.043	1.874	0.061
Zhang et al. (2017)	0.327	0.110	0.012	0.111	0.543	2.973	0.003
	0.344	0.055	0.003	0.235	0.452	6.197	0.000

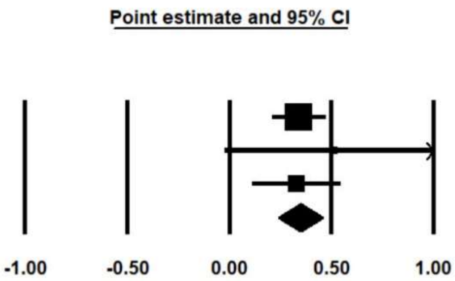

Supplementary Fig. 18. The forest plot of individual studies and the overall outcome for heritability estimate of Fig. Details are provided in Supplementary Fig. 1.

Study name — **Statistics for each study**

Study name	Point estimate	Standard error	Variance	Lower limit	Upper limit	Z-Value	p-Value
Basso et al. (2012)	0.340	0.060	0.004	0.222	0.458	5.667	0.000
Chapuis et al. (2022)- Data set 1	0.490	0.080	0.006	0.333	0.647	6.125	0.000
Chapuis et al. (2022)- Data set 2	0.540	0.120	0.014	0.305	0.775	4.500	0.000
Li et al. (2020)	0.330	0.028	0.001	0.275	0.385	11.828	0.000
Liu et al. (2021)	0.510	0.115	0.013	0.285	0.735	4.440	0.000
Marie-Etancelin et al. (2011)- Data set 1	0.060	0.030	0.001	0.001	0.119	2.000	0.046
Marie-Etancelin et al. (2011)- Data set 2	0.050	0.020	0.000	0.011	0.089	2.500	0.012
Zeng et al. (2018)- Data set 1	0.220	0.060	0.004	0.102	0.338	3.667	0.000
Zeng et al. (2018)- Data set 2	0.240	0.060	0.004	0.122	0.358	4.000	0.000
	0.288	0.059	0.003	0.173	0.404	4.913	0.000

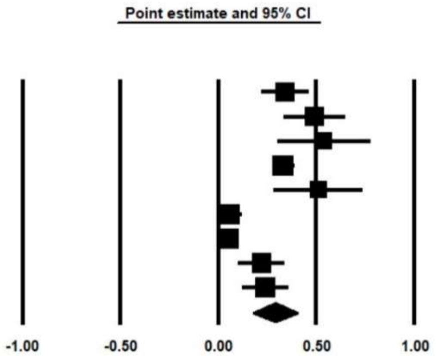

Supplementary Fig. 19. The forest plot of individual studies and the overall outcome for heritability estimate of FI_{gd}. Details are provided in Supplementary Fig. 1.

Study name — **Statistics for each study**

Study name	Point estimate	Standard error	Variance	Lower limit	Upper limit	Z-Value	p-Value
Basso et al. (2012)	0.320	0.100	0.010	0.124	0.516	3.200	0.001
Brun and Lazrul (2003)- Data set 1	0.150	0.030	0.001	0.091	0.209	5.000	0.000
Brun and Lazrul (2003)- Data set 2	0.360	0.030	0.001	0.301	0.419	12.000	0.000
	0.271	0.085	0.007	0.106	0.437	3.210	0.001

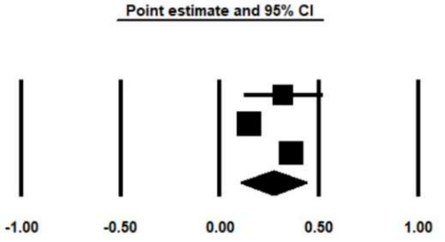

Supplementary Fig. 20. The forest plot of individual studies and the overall outcome for heritability estimate of FER. Details are provided in Supplementary Fig. 1.

Study name	Point estimate	Standard error	Variance	Lower limit	Upper limit	Z-Value	p-Value
		Statistics for each study					
Basso et al. (2012)	0.240	0.110	0.012	0.024	0.456	2.182	0.029
Brun and Lazrul (2003)- Data set 1	0.160	0.030	0.001	0.101	0.219	5.333	0.000
Brun and Lazrul (2003)- Data set 2	0.320	0.040	0.002	0.242	0.398	8.000	0.000
	0.238	0.064	0.004	0.112	0.364	3.707	0.000

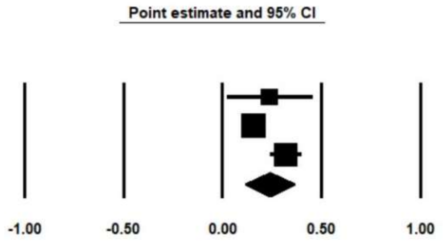

Supplementary Fig. 21. The forest plot of individual studies and the overall outcome for heritability estimate of HR. Details are provided in Supplementary Fig. 1.

Study name	Point estimate	Standard error	Variance	Lower limit	Upper limit	Z-Value	p-Value
		Statistics for each study					
Cai et al. (2023)	0.460	0.070	0.005	0.323	0.597	6.571	0.000
Mucha et al. (2014)	0.510	0.160	0.026	0.196	0.824	3.188	0.001
Wähner et al. (2017)	0.420	0.067	0.005	0.288	0.552	6.225	0.000
Xu et al. (2011)	0.380	0.056	0.003	0.270	0.490	6.764	0.000
	0.419	0.036	0.001	0.349	0.489	11.692	0.000

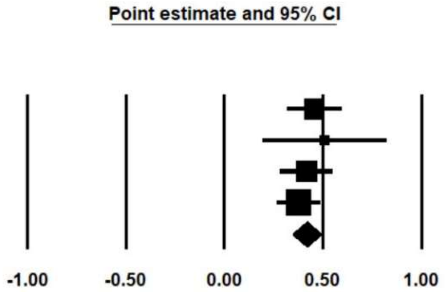

Supplementary Fig. 22. The forest plot of individual studies and the overall outcome for heritability estimate of LMW. Details are provided in Supplementary Fig. 1.

Study name	Point estimate	Standard error	Variance	Lower limit	Upper limit	Z-Value	p-Value
		Statistics for each study					
Cai et al. (2023)	0.320	0.060	0.004	0.202	0.438	5.333	0.000
Lazrul (2002)	0.104	0.029	0.001	0.048	0.160	3.634	0.000
Marie-Etancelin et al. (2011)- Data set 1	0.150	0.030	0.001	0.091	0.209	5.000	0.000
Marie-Etancelin et al. (2011)- Data set 2	0.080	0.030	0.001	0.021	0.139	2.667	0.008
Mucha et al. (2014)	0.290	0.140	0.020	0.016	0.564	2.071	0.038
	0.156	0.036	0.001	0.085	0.227	4.310	0.000

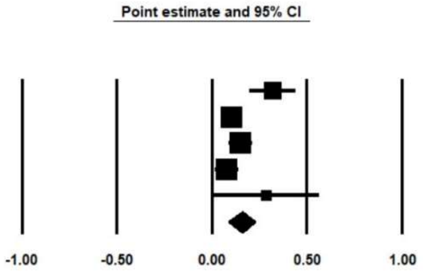

Supplementary Fig. 23. The forest plot of individual studies and the overall outcome for heritability estimate of LW. Details are provided in Supplementary Fig. 1.

Study name	Point estimate	Standard error	Variance	Lower limit	Upper limit	Z-Value	p-Value
Chapuis et al. (2022)- Data set 1	0.480	0.070	0.005	0.343	0.617	6.857	0.000
Chapuis et al. (2022)- Data set 2	0.380	0.110	0.012	0.164	0.596	3.455	0.001
Li et al. (2020)	0.650	0.028	0.001	0.595	0.705	23.257	0.000
	0.526	0.084	0.007	0.362	0.690	6.287	0.000

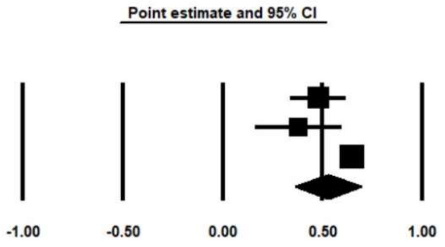

Supplementary Fig. 24. The forest plot of individual studies and the overall outcome for heritability estimate of MD. Details are provided in Supplementary Fig. 1.

Study name	Point estimate	Standard error	Variance	Lower limit	Upper limit	Z-Value	p-Value
Chapuis et al. (2022)- Data set 1	0.630	0.070	0.005	0.493	0.767	9.000	0.000
Chapuis et al. (2022)- Data set 2	0.670	0.110	0.012	0.454	0.886	6.091	0.000
Li et al. (2020)	0.620	0.028	0.001	0.565	0.675	22.183	0.000
	0.624	0.025	0.001	0.574	0.673	24.698	0.000

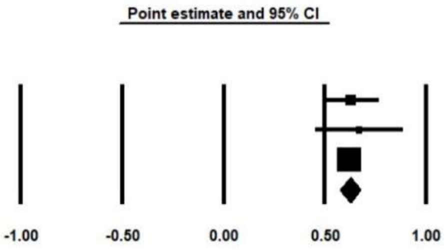

Supplementary Fig. 25. The forest plot of individual studies and the overall outcome for heritability estimate of MFI. Details are provided in Supplementary Fig. 1.

Study name	Point estimate	Standard error	Variance	Lower limit	Upper limit	Z-Value	p-Value
Chapuis et al. (2022)- Data set 1	0.570	0.070	0.005	0.433	0.707	8.143	0.000
Chapuis et al. (2022)- Data set 2	0.540	0.110	0.012	0.324	0.756	4.909	0.000
Li et al. (2020)	0.570	0.028	0.001	0.515	0.625	20.394	0.000
	0.568	0.025	0.001	0.519	0.618	22.500	0.000

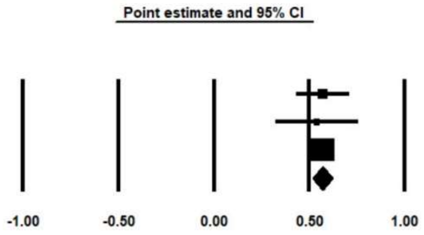

Supplementary Fig. 26. The forest plot of individual studies and the overall outcome for heritability estimate of NMD. Details are provided in Supplementary Fig. 1.

Study name	Point estimate	Standard error	Variance	Lower limit	Upper limit	Z-Value	p-Value
Basso et al. (2012)	0.240	0.110	0.012	0.024	0.456	2.182	0.029
Drouilhet et al. (2014)	0.830	0.420	0.176	0.007	1.653	1.976	0.048
Li et al. (2020)	0.320	0.124	0.015	0.077	0.563	2.578	0.010
Zeng et al. (2018)- Data set 1	0.270	0.060	0.004	0.152	0.388	4.500	0.000
Zeng et al. (2018)- Data set 2	0.240	0.050	0.003	0.142	0.338	4.800	0.000
Zhang et al. (2017)	0.410	0.180	0.032	0.057	0.763	2.278	0.023
	0.266	0.034	0.001	0.199	0.332	7.799	0.000

(Statistics for each study)

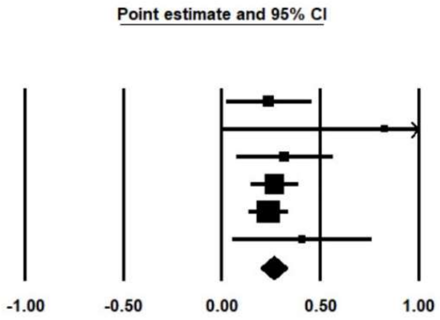

Supplementary Fig. 27. The forest plot of individual studies and the overall outcome for heritability estimate of RFI. Details are provided in Supplementary Fig. 1.

Study name	Point estimate	Standard error	Variance	Lower limit	Upper limit	Z-Value	p-Value
Cai et al. (2023)	0.550	0.080	0.006	0.393	0.707	6.875	0.000
Zhang et al. (2022)- Data set 1	0.540	0.090	0.008	0.364	0.716	6.000	0.000
Zhang et al. (2022)- Data set 2	0.340	0.090	0.008	0.164	0.516	3.778	0.000
	0.480	0.067	0.005	0.348	0.612	7.133	0.000

(Statistics for each study)

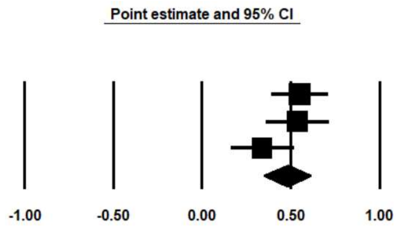

Supplementary Fig. 28. The forest plot of individual studies and the overall outcome for heritability estimate of SFP. Details are provided in Supplementary Fig. 1.

Study name	Point estimate	Standard error	Variance	Lower limit	Upper limit	Z-Value	p-Value
Cai et al. (2023)	0.600	0.080	0.006	0.443	0.757	7.500	0.000
Zhang et al. (2022)- Data set 1	0.370	0.110	0.012	0.154	0.586	3.364	0.001
Zhang et al. (2022)- Data set 2	0.340	0.080	0.006	0.183	0.497	4.250	0.000
	0.442	0.088	0.008	0.268	0.615	4.990	0.000

(Statistics for each study)

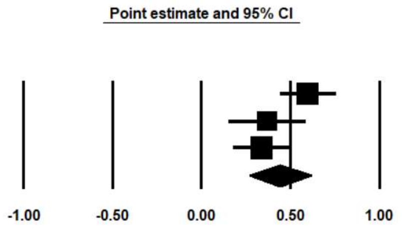

Supplementary Fig. 29. The forest plot of individual studies and the overall outcome for heritability estimate of SFW. Details are provided in Supplementary Fig. 1.

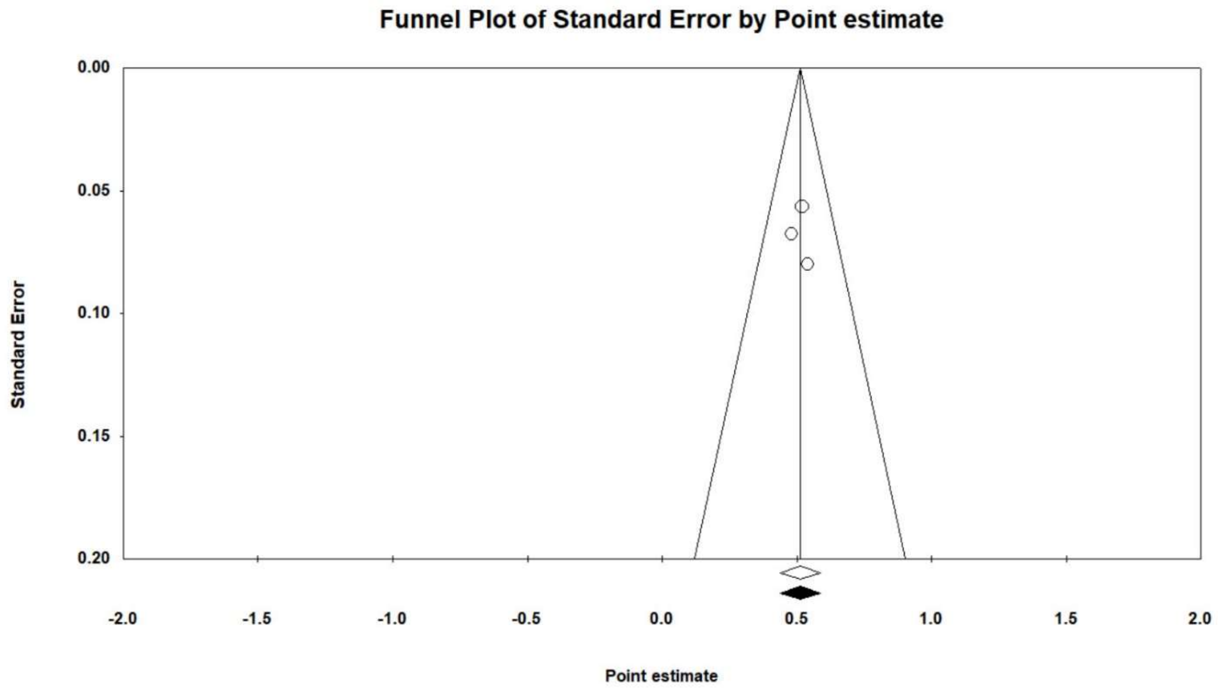

Supplementary Fig. 30. The funnel plot of the heritability estimate for CW. Details are provided in Fig. 2.

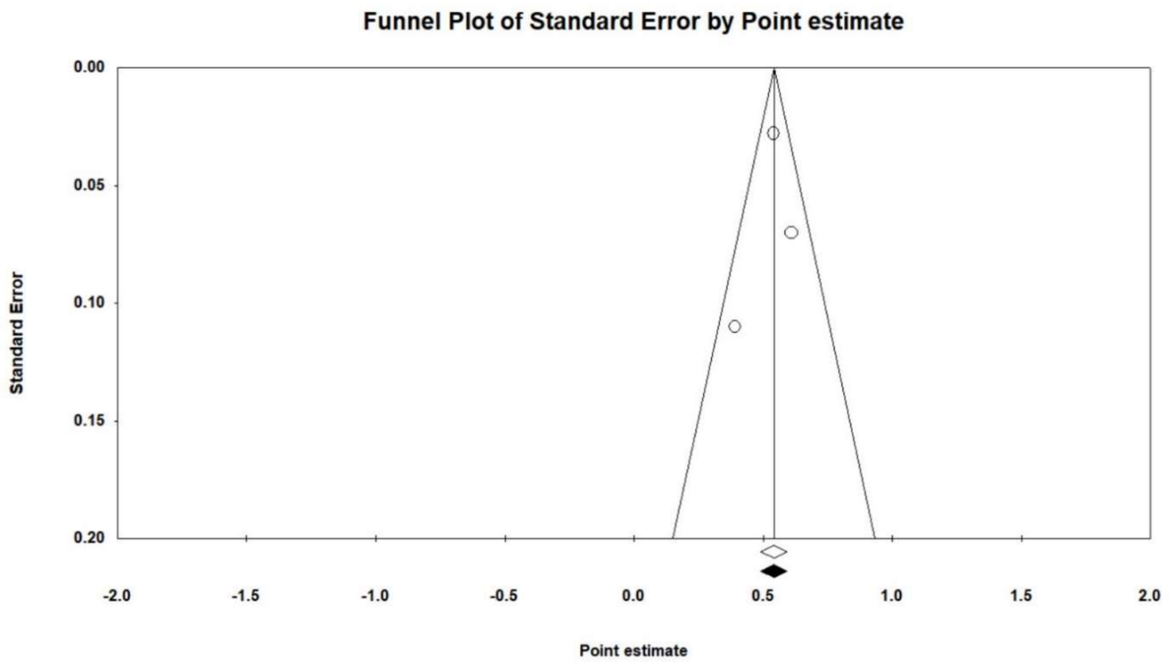

Supplementary Fig. 31. The funnel plot of the heritability estimate for DFR. Details are provided in Fig. 2.

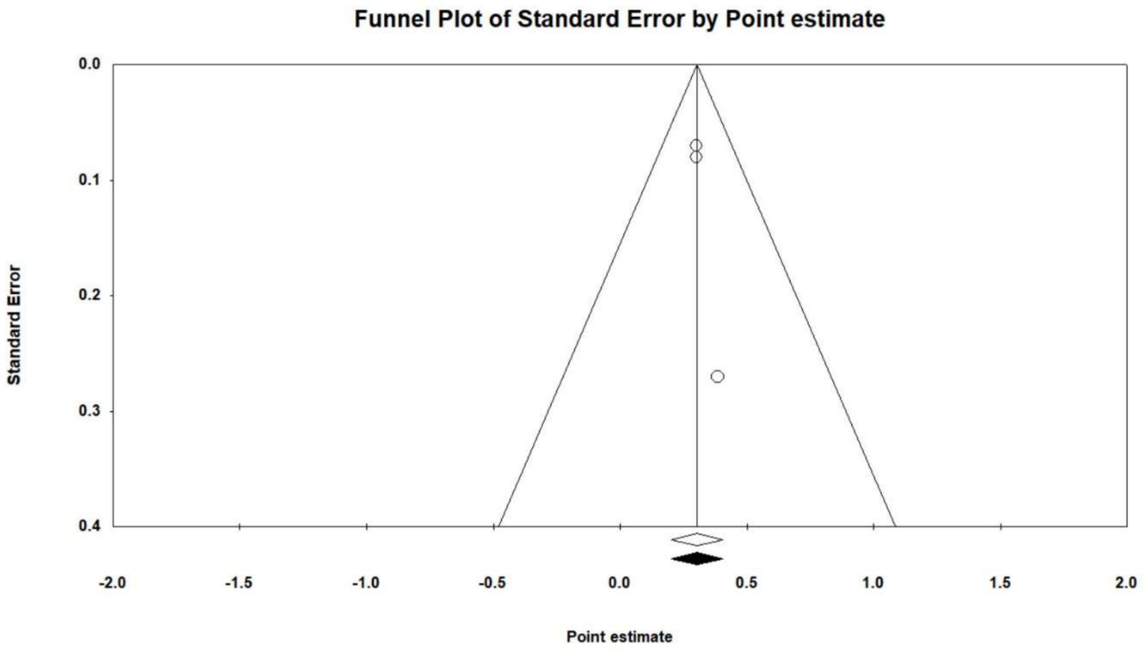

Supplementary Fig. 32. The funnel plot of the heritability estimate for EM_g. Details are provided in Fig. 2.

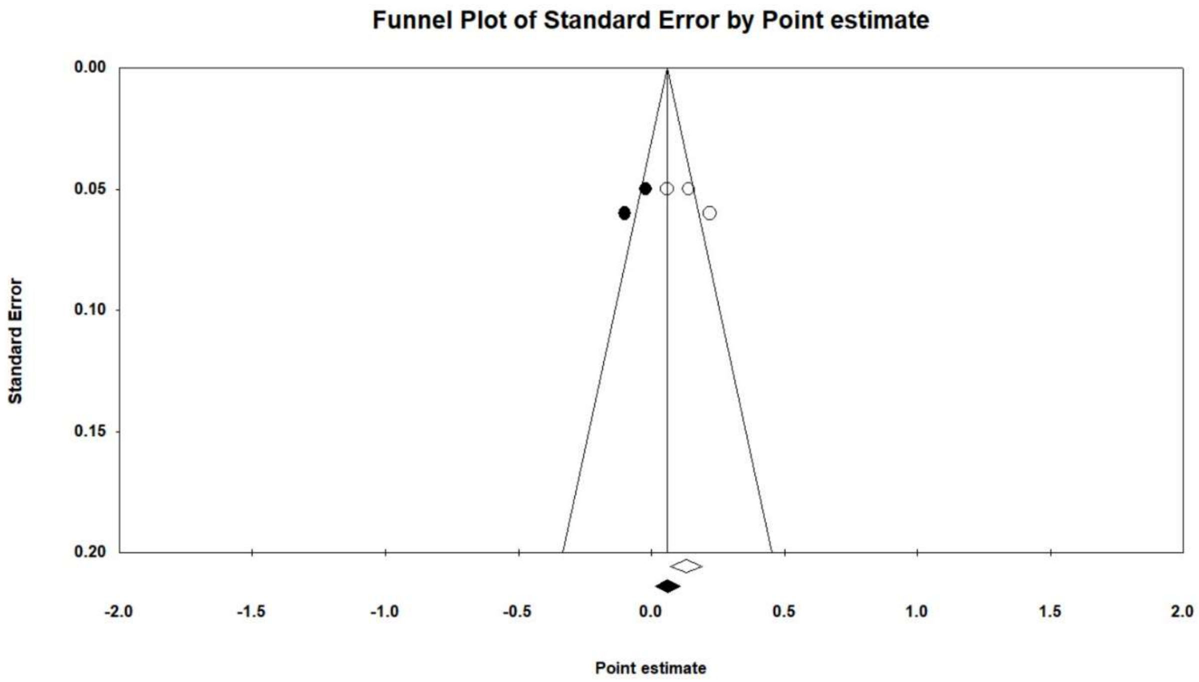

Supplementary Fig. 33. The funnel plot of the heritability estimate for EM_{gd}. Details are provided in Fig. 2.

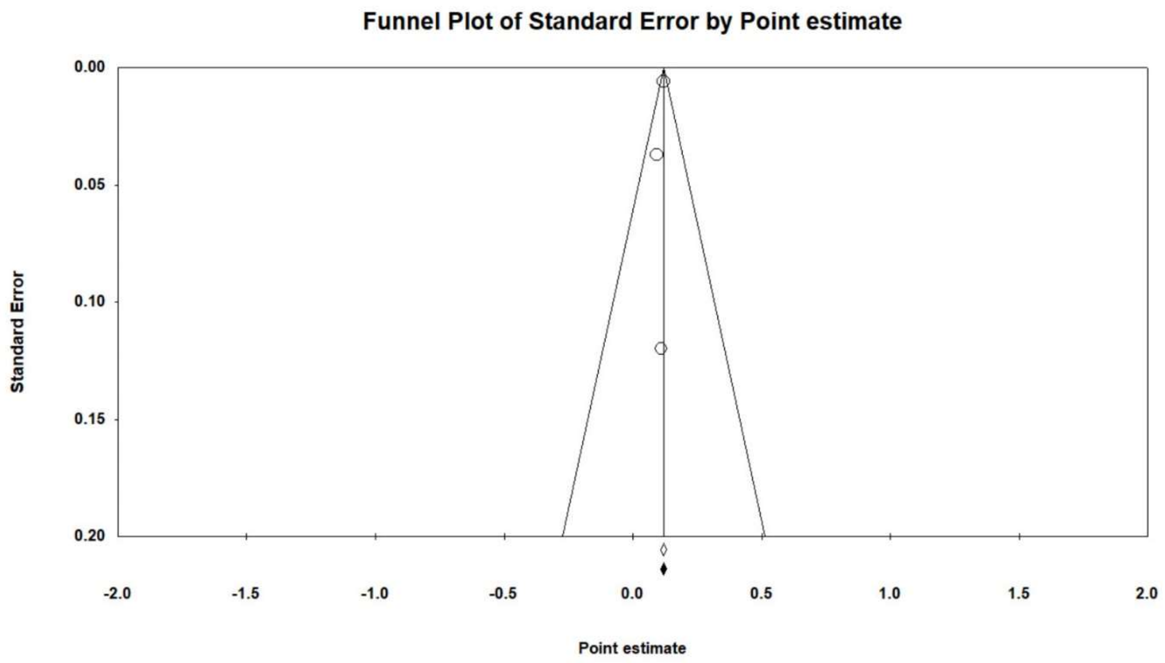

Supplementary Fig. 34. The funnel plot of the heritability estimate for ESS. Details are provided in Fig. 2.

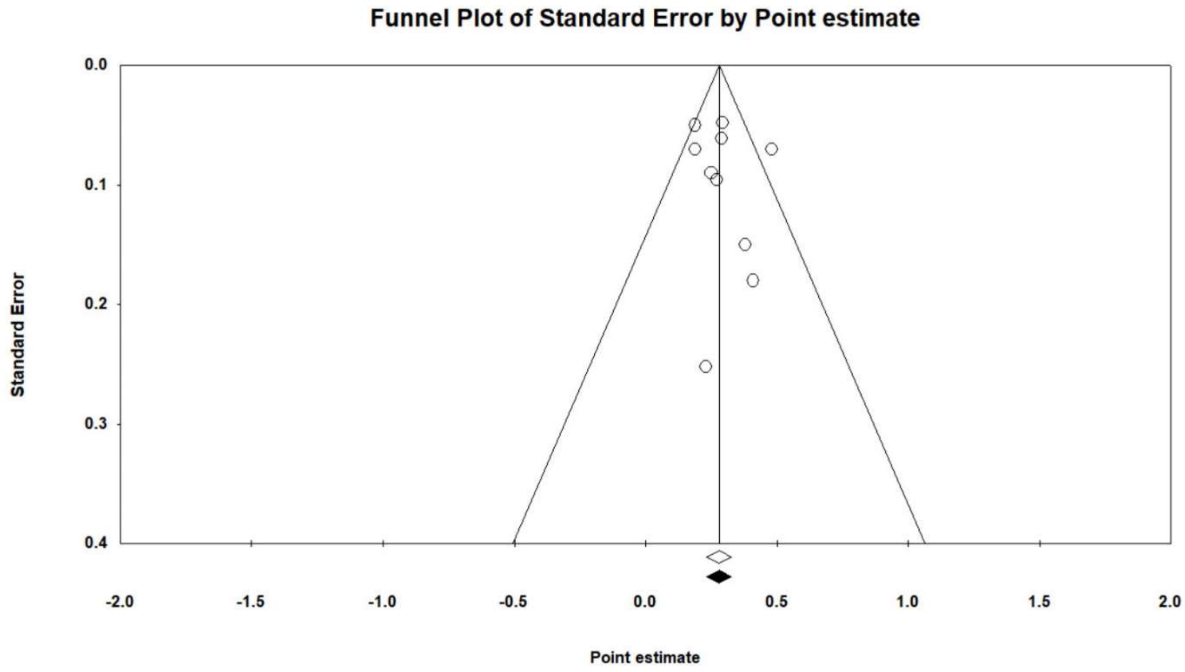

Supplementary Fig. 35. The funnel plot of the heritability estimate for FCR. Details are provided in Fig. 2.

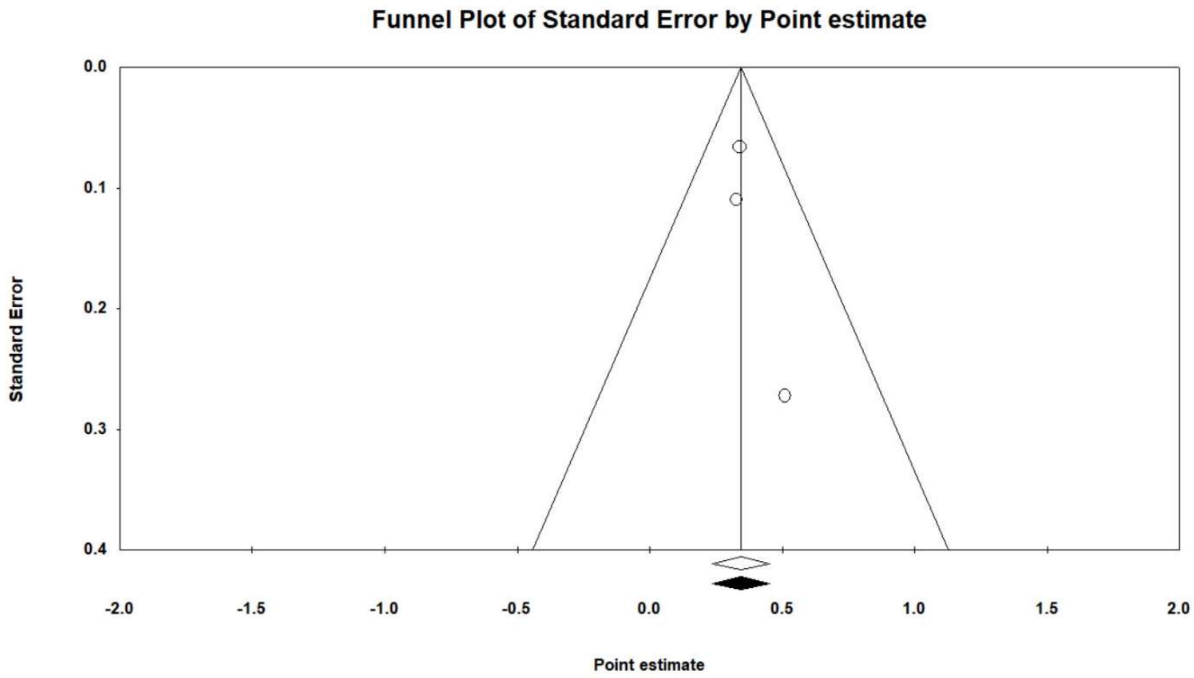

Supplementary Fig. 36. The funnel plot of the heritability estimate for Fig. Details are provided in Fig. 2.

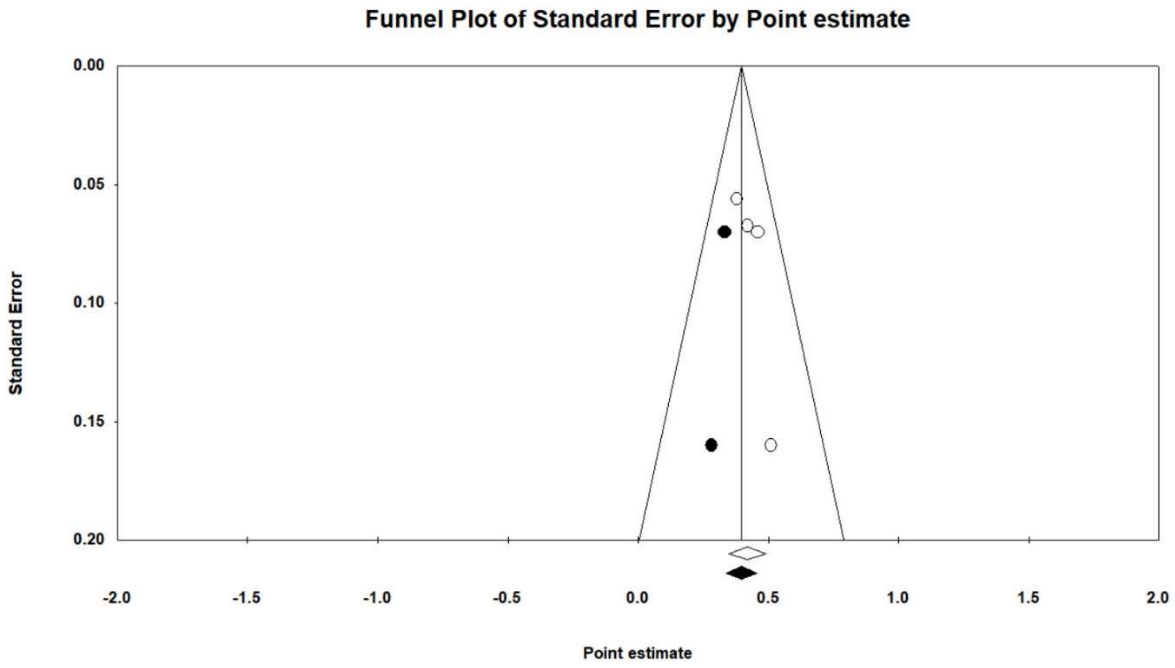

Supplementary Fig. 37. The funnel plot of the heritability estimate for LMW. Details are provided in Fig. 2.

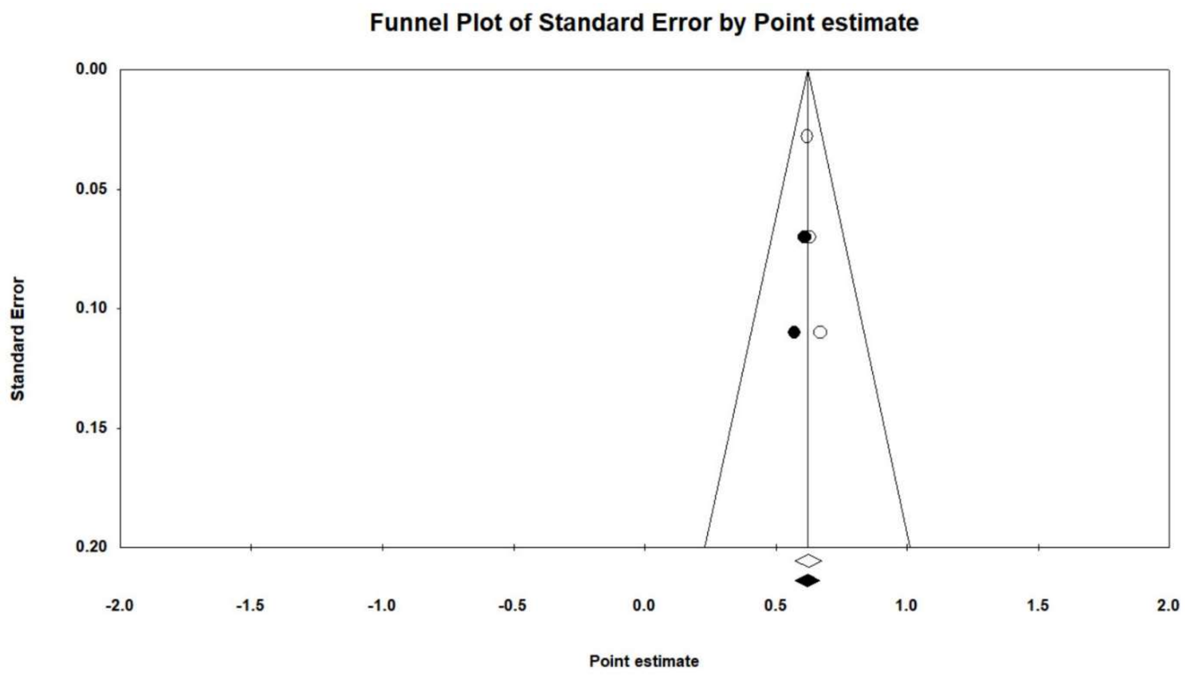

Supplementary Fig. 38. The funnel plot of the heritability estimate for MFI. Details are provided in Fig. 2.

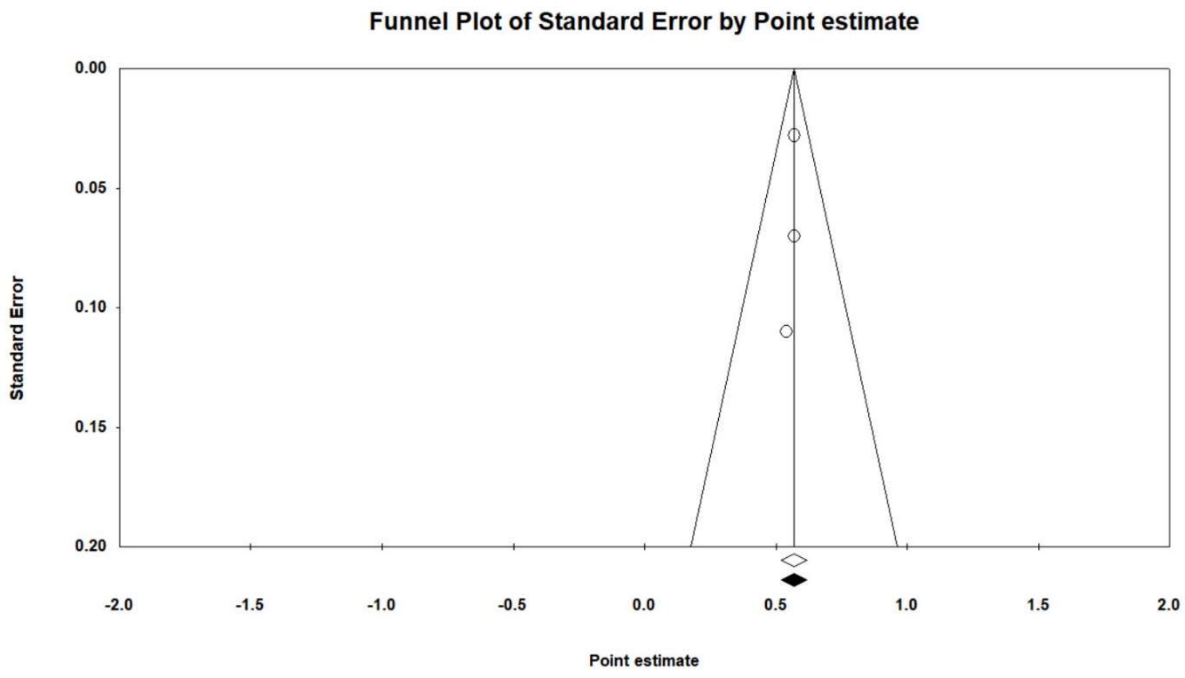

Supplementary Fig. 39. The funnel plot of the heritability estimate for NMD. Details are provided in Fig. 2.

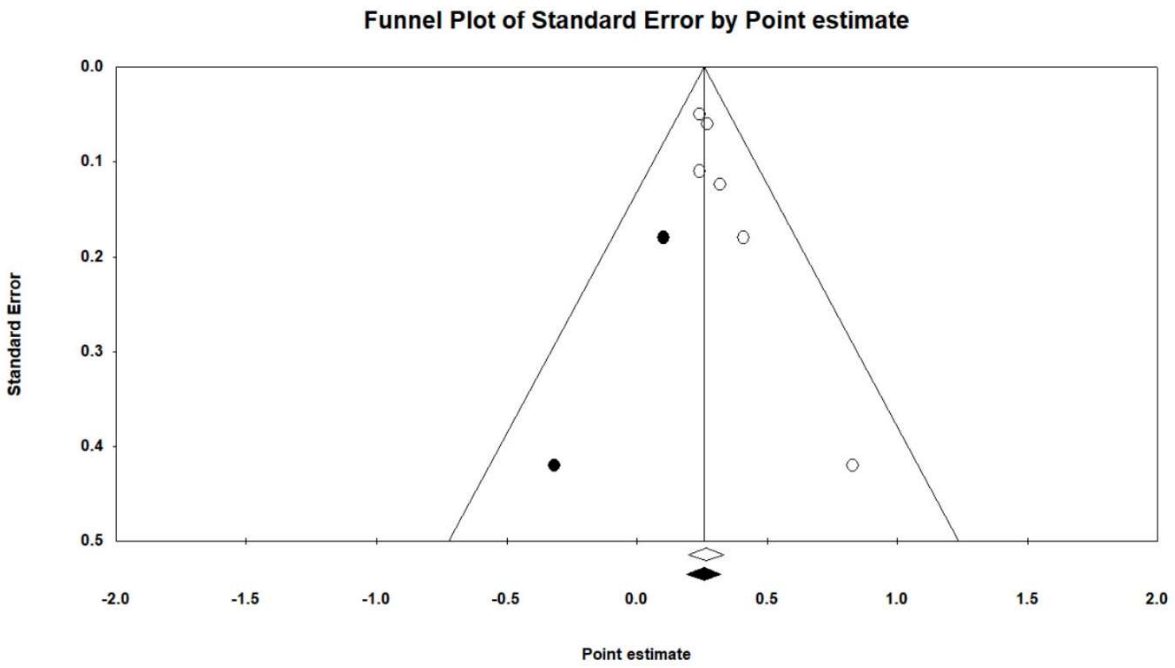

Supplementary Fig. 40. The funnel plot of the heritability estimate for RFI. Details are provided in Fig. 2.

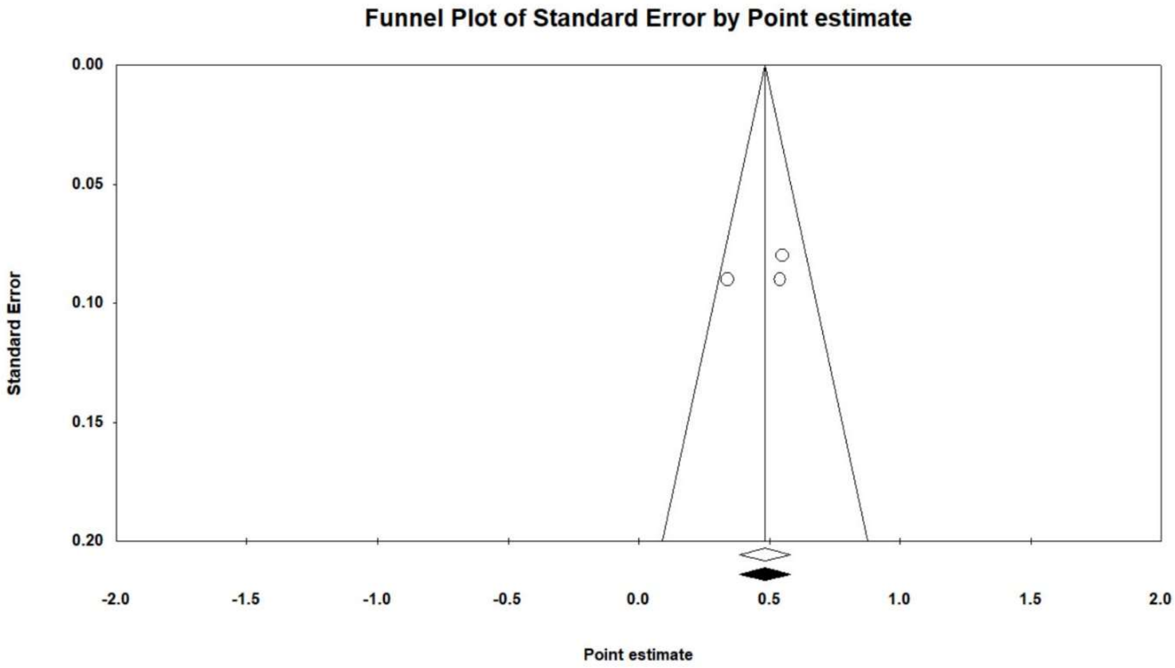

Supplementary Fig. 41. The funnel plot of the heritability estimate for SFP. Details are provided in Fig. 2.

Study name	Correlation	Lower limit	Upper limit	Z-Value	p-Value
Cai et al. (2023)	0.454	0.236	0.629	3.849	0.000
Mucha et al. (2014)	-0.290	-0.705	0.272	-1.013	0.311
Xu et al. (2011)	0.240	0.063	0.402	2.644	0.008
	0.235	-0.068	0.498	1.526	0.127

Statistics for each study

Correlation and 95% CI

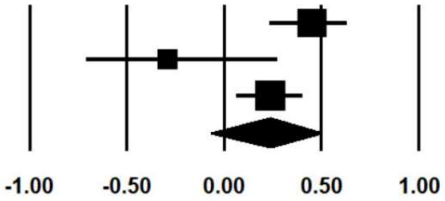

Supplementary Fig. 42. The forest plot of individual studies and the overall outcome for the genetic correlation estimate between BMW-AFW. Details are provided in Supplementary Fig. 1.

Study name	Correlation	Lower limit	Upper limit	Z-Value	p-Value
Cai et al. (2023)	0.548	0.338	0.706	4.582	0.000
Xu et al. (2011)	0.910	0.647	0.979	3.953	0.000
Xu et al. (2018)	0.660	0.607	0.707	17.614	0.000
	0.664	0.495	0.784	6.094	0.000

Statistics for each study

Correlation and 95% CI

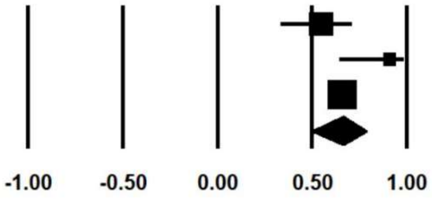

Supplementary Fig. 43. The forest plot of individual studies and the overall outcome for the genetic correlation estimate between BMW-BMP. Details are provided in Supplementary Fig. 1.

Study name	Correlation	Lower limit	Upper limit	Z-Value	p-Value
Cai et al. (2023)	0.636	0.442	0.773	5.327	0.000
Mucha et al. (2014)	0.880	0.533	0.974	3.449	0.001
Wähner et al. (2017)	0.520	0.381	0.636	6.457	0.000
Xu et al. (2011)	0.280	0.171	0.383	4.890	0.000
	0.542	0.305	0.716	4.075	0.000

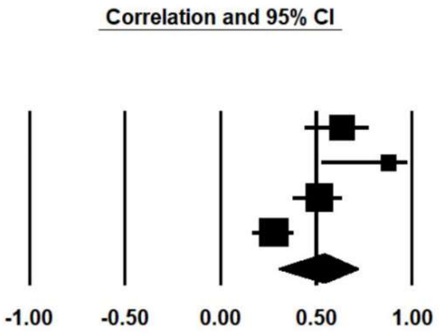

Supplementary Fig. 44. The forest plot of individual studies and the overall outcome for the genetic correlation estimate between BMW-LMW. Details are provided in Supplementary Fig. 1.

Study name	Correlation	Lower limit	Upper limit	Z-Value	p-Value
Brun and Lazrul (2003)- Data set 1	-0.140	-0.217	-0.061	-3.454	0.001
Brun and Lazrul (2003)- Data set 2	-0.070	-0.128	-0.011	-2.326	0.020
Brun and Lazrul (2003)- Data set 3	0.080	0.001	0.158	1.991	0.046
Cheng et al. (1995)- Data set 1	0.065	-0.032	0.161	1.314	0.189
Cheng et al. (1995)- Data set 2	0.058	-0.039	0.154	1.173	0.241
Hu et al. (2004)- Data set 1	0.410	0.286	0.520	6.040	0.000
Hu et al. (2004)- Data set 2	0.460	0.335	0.569	6.535	0.000
Hu et al. (2004)- Data set 3	0.470	0.253	0.642	3.974	0.000
Hu et al. (2004)- Data set 4	0.710	0.598	0.795	8.799	0.000
Lin et al. (2016)- Data set 1	-0.166	-0.684	0.463	-0.491	0.624
Lin et al. (2016)- Data set 2	0.491	-0.226	0.863	1.373	0.170
	0.230	0.076	0.373	2.907	0.004

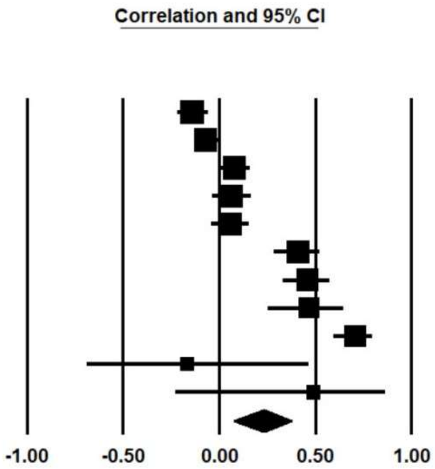

Supplementary Fig. 45. The forest plot of individual studies and the overall outcome for the genetic correlation estimate between BW-AFE. Details are provided in Supplementary Fig. 1.

Study name	Correlation	Lower limit	Upper limit	Z-Value	p-Value
Cai et al. (2024)-Data set 1	0.150	-0.235	0.494	0.759	0.448
Cai et al. (2024)-Data set 2	0.640	-0.221	0.940	1.512	0.131
Cai et al. (2024)-Data set 3	0.620	-0.186	0.927	1.556	0.120
Cai et al. (2024)-Data set 4	0.550	0.071	0.823	2.215	0.027
Duggan et al. (2017)- Data set 1	0.452	0.129	0.688	2.674	0.008
Duggan et al. (2017)- Data set 2	0.230	0.002	0.435	1.980	0.048
Wähner et al. (2017)- Data set 1	0.400	0.006	0.687	1.988	0.047
Wähner et al. (2017)- Data set 2	0.470	0.060	0.744	2.220	0.026
Xu et al. (2011)	0.570	0.212	0.793	2.933	0.003
Xu et al. (2018)	0.720	0.714	0.726	137.171	0.000
	0.484	0.256	0.661	3.888	0.000

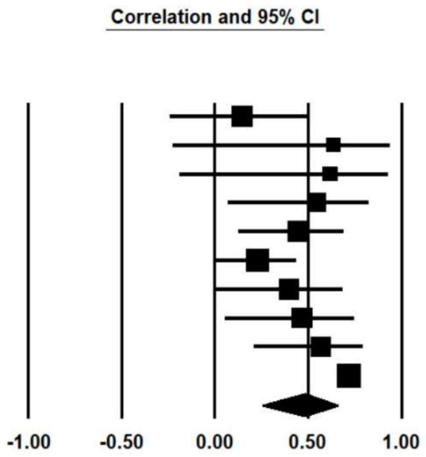

Supplementary Fig. 46. The forest plot of individual studies and the overall outcome for the genetic correlation estimate between BW-BMT. Details are provided in Supplementary Fig. 1.

Study name	Correlation	Lower limit	Upper limit	Z-Value	p-Value
Mucha et al. (2014)	0.690	0.313	0.879	3.173	0.002
Wähner et al. (2017)- Data set 1	0.440	0.284	0.573	5.129	0.000
Wähner et al. (2017)- Data set 2	0.710	0.533	0.828	5.926	0.000
Xu et al. (2011)	0.740	0.593	0.839	6.956	0.000
Xu et al. (2018)	0.830	0.778	0.871	15.788	0.000
	0.702	0.498	0.832	5.273	0.000

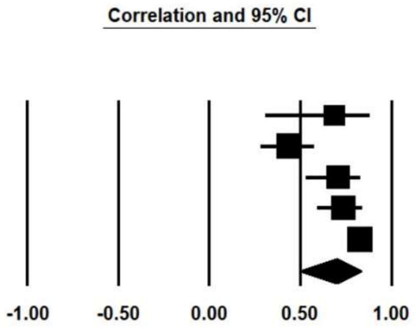

Supplementary Fig. 47. The forest plot of individual studies and the overall outcome for the genetic correlation estimate between BW-BMW. Details are provided in Supplementary Fig. 1.

Study name	Correlation	Lower limit	Upper limit	Z-Value	p-Value
Cai et al. (2024)-Data set 1	0.270	0.007	0.498	2.009	0.044
Cai et al. (2024)-Data set 10	0.999	-1.000	1.000	0.039	0.969
Cai et al. (2024)-Data set 11	0.110	-0.266	0.457	0.566	0.572
Cai et al. (2024)-Data set 12	0.450	0.011	0.744	2.004	0.045
Cai et al. (2024)-Data set 13	0.780	0.080	0.965	2.122	0.034
Cai et al. (2024)-Data set 14	0.230	-0.155	0.554	1.175	0.240
Cai et al. (2024)-Data set 15	0.999	-1.000	1.000	0.040	0.968
Cai et al. (2024)-Data set 16	0.870	-0.186	0.993	1.717	0.086
Cai et al. (2024)-Data set 2	0.980	-1.000	1.000	0.467	0.640
Cai et al. (2024)-Data set 3	0.870	-0.186	0.993	1.717	0.086
Cai et al. (2024)-Data set 4	0.999	-1.000	1.000	0.059	0.953
Cai et al. (2024)-Data set 5	0.450	0.006	0.746	1.984	0.047
Cai et al. (2024)-Data set 6	0.999	-1.000	1.000	0.039	0.969
Cai et al. (2024)-Data set 7	0.990	-1.000	1.000	0.270	0.787
Cai et al. (2024)-Data set 8	0.310	-0.105	0.633	1.474	0.141
Cai et al. (2024)-Data set 9	0.970	-1.000	1.000	0.629	0.529
Drouilhet et al. (2014)	0.760	0.114	0.954	2.215	0.027
Li et al. (2020)- Data set 1	0.250	0.132	0.361	4.099	0.000
Li et al. (2020)- Data set 2	0.910	0.697	0.975	4.495	0.000
Wähner et al. (2017)- Data set 1	0.330	0.084	0.538	2.594	0.009
Wähner et al. (2017)- Data set 2	0.970	-0.948	1.000	1.050	0.294
Zeng et al. (2018)- Data set 1	-0.040	-0.157	0.078	-0.666	0.505
Zeng et al. (2018)- Data set 2	0.020	-0.039	0.079	0.666	0.505
Zhang et al. (2017)	0.920	0.514	0.989	3.051	0.002
	0.328	0.191	0.451	4.551	0.000

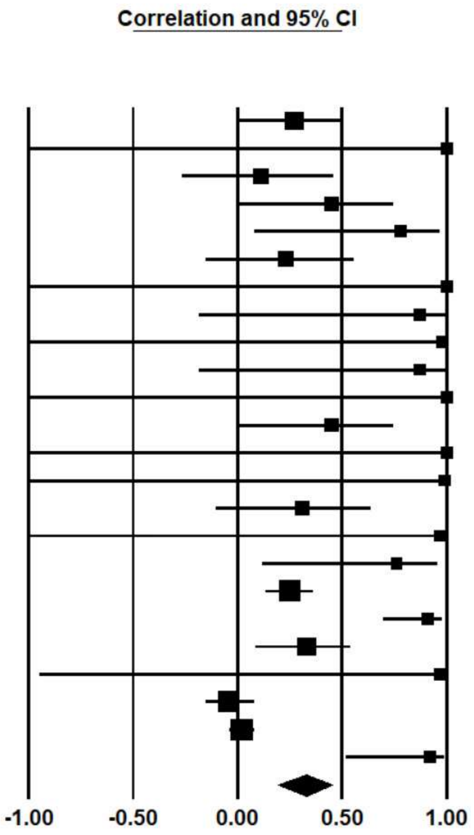

Supplementary Fig. 48. The forest plot of individual studies and the overall outcome for the genetic correlation estimate between BW-BWG. Details are provided in Supplementary Fig. 1.

Study name	Correlation	Lower limit	Upper limit	Z-Value	p-Value
El-Deghadi et al. (2022)- Data set 1	0.500	0.462	0.536	21.683	0.000
El-Deghadi et al. (2022)- Data set 2	0.580	0.554	0.605	33.816	0.000
El-Deghadi et al. (2022)- Data set 3	0.870	0.860	0.879	64.814	0.000
El-Deghadi et al. (2022)- Data set 4	0.820	0.806	0.833	54.139	0.000
Zeng et al. (2018)- Data set 1	0.030	-0.068	0.127	0.600	0.549
Zeng et al. (2018)- Data set 2	0.240	0.099	0.372	3.295	0.001
	0.582	0.313	0.765	3.811	0.000

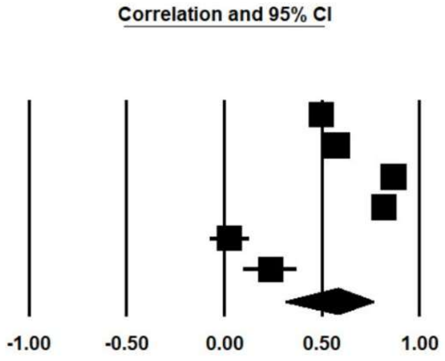

Supplementary Fig. 49. The forest plot of individual studies and the overall outcome for the genetic correlation estimate between BW-EM. Details are provided in Supplementary Fig. 1.

Study name	Correlation	Lower limit	Upper limit	Z-Value	p-Value
Boonlert et al. (2005)	0.059	0.039	0.079	5.886	0.000
Brun and Lazrul (2003)- Data set 1	0.360	0.194	0.506	4.101	0.000
Brun and Lazrul (2003)- Data set 2	0.400	0.276	0.511	5.931	0.000
Brun and Lazrul (2003)- Data set 3	0.030	-0.087	0.147	0.500	0.617
Cheng et al. (1995)- Data set 1	0.006	-0.112	0.124	0.099	0.921
Cheng et al. (1995)- Data set 2	-0.058	-0.175	0.061	-0.956	0.339
Cheng et al. (1995)- Data set 3	-0.090	-0.206	0.029	-1.483	0.138
Cheng et al. (1995)- Data set 4	-0.148	-0.264	-0.028	-2.417	0.016
Damayanti et al. (2019)- Data set 1	-0.350	-0.801	0.355	-0.972	0.331
Damayanti et al. (2019)- Data set 2	-0.810	-0.999	0.923	-0.807	0.419
El-Deghadi et al. (2022)- Data set 1	0.520	0.323	0.674	4.672	0.000
El-Deghadi et al. (2022)- Data set 2	0.570	0.383	0.712	5.204	0.000
El-Deghadi et al. (2022)- Data set 3	0.710	0.651	0.761	15.713	0.000
El-Deghadi et al. (2022)- Data set 4	0.820	0.781	0.852	21.054	0.000
Hu et al. (2004)- Data set 1	-0.320	-0.414	-0.219	-5.954	0.000
Hu et al. (2004)- Data set 10	-0.300	-0.413	-0.178	-4.694	0.000
Hu et al. (2004)- Data set 11	-0.200	-0.386	0.001	-1.946	0.052
Hu et al. (2004)- Data set 12	-0.510	-0.634	-0.360	-5.948	0.000
Hu et al. (2004)- Data set 13	-0.340	-0.452	-0.218	-5.219	0.000
Hu et al. (2004)- Data set 14	-0.280	-0.393	-0.159	-4.419	0.000
Hu et al. (2004)- Data set 15	-0.220	-0.388	-0.038	-2.365	0.018
Hu et al. (2004)- Data set 16	-0.500	-0.624	-0.351	-5.885	0.000
Hu et al. (2004)- Data set 2	-0.410	-0.520	-0.286	-6.040	0.000
Hu et al. (2004)- Data set 3	-0.450	-0.623	-0.234	-3.865	0.000
Hu et al. (2004)- Data set 4	-0.700	-0.785	-0.588	-8.846	0.000
Hu et al. (2004)- Data set 5	-0.330	-0.424	-0.229	-6.110	0.000
Hu et al. (2004)- Data set 6	-0.340	-0.434	-0.239	-6.263	0.000
Hu et al. (2004)- Data set 7	-0.320	-0.484	-0.134	-3.308	0.001
Hu et al. (2004)- Data set 8	-0.600	-0.705	-0.469	-7.394	0.000
Hu et al. (2004)- Data set 9	-0.320	-0.432	-0.198	-4.961	0.000
Lin et al. (2016)- Data set 1	0.318	-0.264	0.730	1.077	0.282
Lin et al. (2016)- Data set 2	0.186	-0.349	0.629	0.668	0.504
Lin et al. (2016)- Data set 3	0.364	-0.267	0.777	1.141	0.254
Lin et al. (2016)- Data set 4	0.427	-0.111	0.772	1.574	0.115
Lin et al. (2016)- Data set 5	0.410	-0.236	0.805	1.263	0.207
Lin et al. (2016)- Data set 6	0.545	-0.022	0.847	1.893	0.058
	-0.048	-0.181	0.086	-0.702	0.483

Statistics for each study

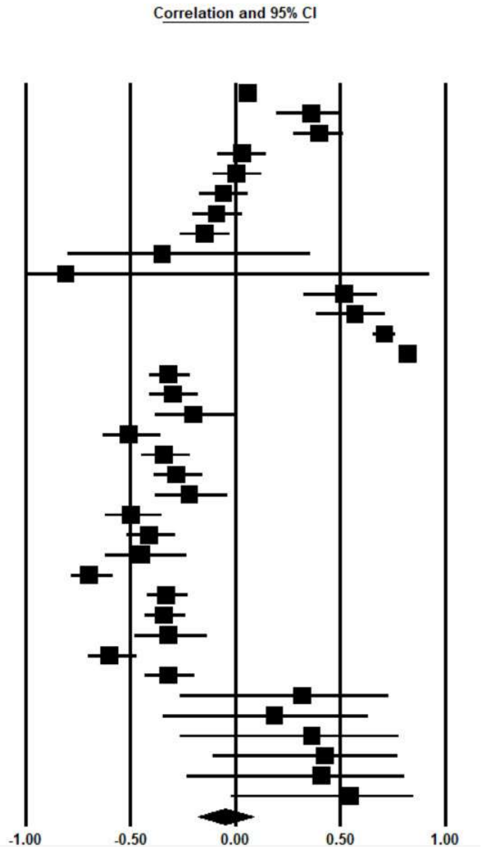

Supplementary Fig. 50. The forest plot of individual studies and the overall outcome for the genetic correlation estimate between BW-EN. Details are provided in Supplementary Fig. 1.

Study name	Correlation	Lower limit	Upper limit	Z-Value	p-Value
Boonlert et al. (2005)	0.607	0.175	0.843	2.616	0.009
Cheng et al. (1995)- Data set 1	0.617	0.509	0.706	8.872	0.000
Cheng et al. (1995)- Data set 2	0.596	0.489	0.686	8.811	0.000
Cheng et al. (1995)- Data set 3	0.523	0.415	0.616	8.200	0.000
Cheng et al. (1995)- Data set 4	0.463	0.356	0.558	7.656	0.000
El-Deghadi et al. (2022)- Data set 1	0.570	0.373	0.718	4.968	0.000
El-Deghadi et al. (2022)- Data set 2	0.500	0.307	0.654	4.629	0.000
El-Deghadi et al. (2022)- Data set 3	0.700	0.576	0.793	8.042	0.000
El-Deghadi et al. (2022)- Data set 4	0.820	0.736	0.879	10.527	0.000
Lin et al. (2016)- Data set 1	0.502	0.046	0.785	2.139	0.032
Lin et al. (2016)- Data set 2	0.483	0.031	0.771	2.082	0.037
Lin et al. (2016)- Data set 3	0.879	0.061	0.991	2.051	0.040
Lin et al. (2016)- Data set 4	0.664	0.137	0.898	2.366	0.018
Lin et al. (2016)- Data set 5	0.700	0.262	0.899	2.835	0.005
Lin et al. (2016)- Data set 6	0.780	0.537	0.903	4.600	0.000
Lin et al. (2016)- Data set 7	0.798	0.387	0.945	3.126	0.002
Lin et al. (2016)- Data set 8	0.816	0.502	0.940	3.787	0.000
	0.629	0.557	0.691	13.064	0.000

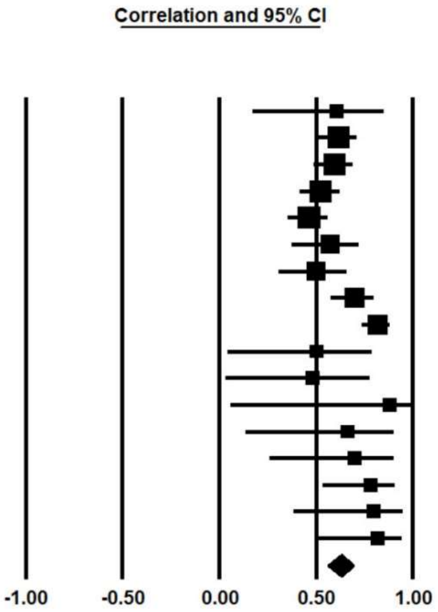

Supplementary Fig. 51. The forest plot of individual studies and the overall outcome for the genetic correlation estimate between BW-EW. Details are provided in Supplementary Fig. 1.

Study name	Correlation	Lower limit	Upper limit	Z-Value	p-Value
Drouilhet et al. (2014)	-0.120	-0.639	0.474	-0.371	0.710
Duggan et al. (2017)- Data set 1	0.609	0.279	0.810	3.296	0.001
Duggan et al. (2017)- Data set 2	0.303	0.039	0.528	2.237	0.025
Li et al. (2020)- Data set 1	0.330	0.132	0.503	3.202	0.001
Li et al. (2020)- Data set 2	-0.090	-0.272	0.098	-0.938	0.348
Zeng et al. (2018)- Data set 1	-0.070	-0.261	0.126	-0.698	0.485
Zeng et al. (2018)- Data set 2	-0.040	-0.157	0.078	-0.666	0.505
Zhang et al. (2017)	-0.640	-0.841	-0.285	-3.197	0.001
	0.055	-0.146	0.251	0.533	0.594

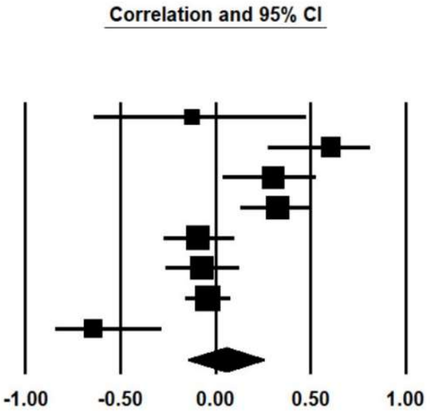

Supplementary Fig. 52. The forest plot of individual studies and the overall outcome for the genetic correlation estimate between BW-FCR. Details are provided in Supplementary Fig. 1.

Study name	Correlation	Lower limit	Upper limit	Z-Value	p-Value
Zeng et al. (2018)- Data set 1	0.510	0.190	0.732	2.974	0.003
Zeng et al. (2018)- Data set 2	0.360	0.106	0.570	2.734	0.006
Zhang et al. (2017)	0.560	0.282	0.751	3.620	0.000
	0.459	0.303	0.592	5.286	0.000

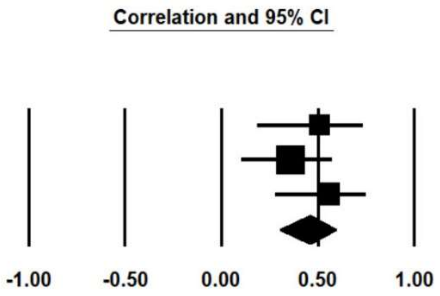

Supplementary Fig. 53. The forest plot of individual studies and the overall outcome for the genetic correlation estimate between BW-FI. Details are provided in Supplementary Fig. 1.

Study name	Correlation	Lower limit	Upper limit	Z-Value	p-Value
Brun and Lazrul (2003)- Data set 1	0.040	-0.155	0.232	0.400	0.689
Brun and Lazrul (2003)- Data set 2	0.210	0.070	0.342	2.911	0.004
Brun and Lazrul (2003)- Data set 3	-0.130	-0.319	0.069	-1.285	0.199
Brun and Lazrul (2003)- Data set 4	0.020	-0.117	0.156	0.286	0.775
Brun and Lazrul (2003)- Data set 5	0.000	-0.117	0.117	0.000	1.000
Brun and Lazrul (2003)- Data set 6	-0.040	-0.157	0.078	-0.666	0.505
	0.021	-0.064	0.107	0.490	0.624

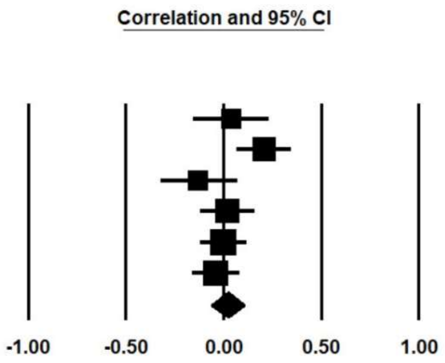

Supplementary Fig. 54. The forest plot of individual studies and the overall outcome for the genetic correlation estimate between BW-FR. Details are provided in Supplementary Fig. 1.

Study name	Correlation	Lower limit	Upper limit	Z-Value	p-Value
Basso et al. (2012)	0.880	-0.268	0.995	1.634	0.102
Zeng et al. (2018)- Data set 1	0.510	0.164	0.744	2.776	0.006
Zeng et al. (2018)- Data set 2	-0.080	-0.138	-0.021	-2.655	0.008
	0.331	-0.279	0.751	1.069	0.285

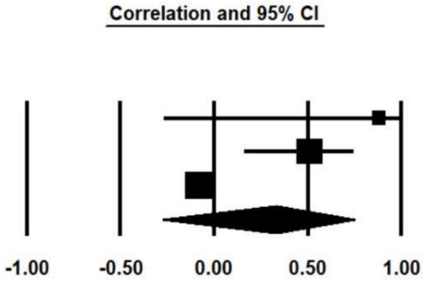

Supplementary Fig. 55. The forest plot of individual studies and the overall outcome for the genetic correlation estimate between BWG-EM. Details are provided in Supplementary Fig. 1.

Study name	Correlation	Lower limit	Upper limit	Z-Value	p-Value
Drouilhet et al. (2014)	-0.600	-0.919	0.193	-1.530	0.126
Li et al. (2020)	-0.280	-0.442	-0.101	-3.025	0.002
Zeng et al. (2018)- Data set 1	-0.240	-0.407	-0.058	-2.563	0.010
Zeng et al. (2018)- Data set 2	0.060	-0.038	0.157	1.197	0.231
Zhang et al. (2017)	-0.800	-0.935	-0.462	-3.595	0.000
	-0.301	-0.532	-0.029	-2.163	0.031

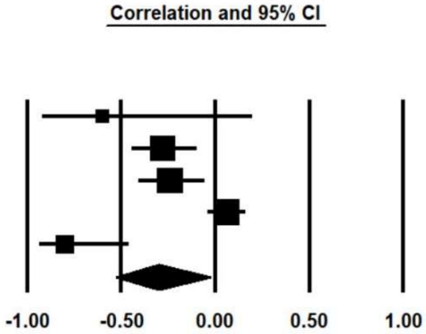

Supplementary Fig. 56. The forest plot of individual studies and the overall outcome for the genetic correlation estimate between BWG-FCR. Details are provided in Supplementary Fig. 1.

Study name	Correlation	Lower limit	Upper limit	Z-Value	p-Value
Basso et al. (2012)	-0.650	-0.993	0.851	-0.746	0.456
Drouilhet et al. (2014)	-0.160	-0.803	0.655	-0.335	0.738
Li et al. (2020)	0.040	-0.247	0.321	0.268	0.788
Zeng et al. (2018)- Data set 1	-0.210	-0.462	0.074	-1.456	0.146
Zeng et al. (2018)- Data set 2	-0.150	-0.284	-0.011	-2.111	0.035
Zhang et al. (2017)	-0.050	-0.464	0.382	-0.217	0.828
	-0.128	-0.235	-0.017	-2.268	0.023

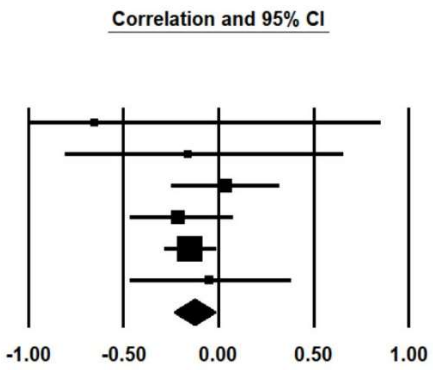

Supplementary Fig. 57. The forest plot of individual studies and the overall outcome for the genetic correlation estimate between BWG-RFI. Details are provided in Supplementary Fig. 1.

Study name	Correlation	Lower limit	Upper limit	Z-Value	p-Value
Brun and Lazrul (2003)- Data set 1	0.190	0.030	0.341	2.317	0.020
Brun and Lazrul (2003)- Data set 2	0.330	0.208	0.442	5.092	0.000
Brun and Lazrul (2003)- Data set 3	0.340	0.153	0.503	3.480	0.001
Brun and Lazrul (2003)- Data set 4	-0.020	-0.175	0.136	-0.250	0.803
Brun and Lazrul (2003)- Data set 5	0.360	0.216	0.489	4.686	0.000
Brun and Lazrul (2003)- Data set 6	0.040	-0.116	0.195	0.499	0.617
	0.210	0.074	0.338	3.001	0.003

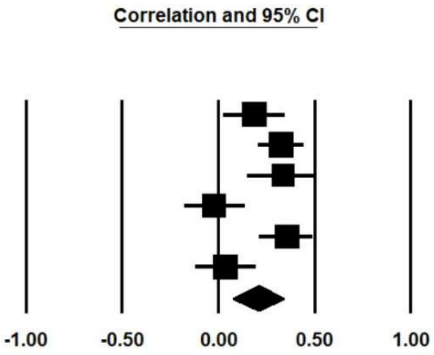

Supplementary Fig. 58. The forest plot of individual studies and the overall outcome for the genetic correlation estimate between BW-HR. Details are provided in Supplementary Fig. 1.

Study name	Correlation	Lower limit	Upper limit	Z-Value	p-Value
Brun and Lazrul (2003)- Data set 1	0.190	0.030	0.341	2.317	0.020
Brun and Lazrul (2003)- Data set 2	0.330	0.208	0.442	5.092	0.000
Brun and Lazrul (2003)- Data set 3	0.340	0.153	0.503	3.480	0.001
Brun and Lazrul (2003)- Data set 4	-0.020	-0.175	0.136	-0.250	0.803
Brun and Lazrul (2003)- Data set 5	0.360	0.216	0.489	4.686	0.000
Brun and Lazrul (2003)- Data set 6	0.040	-0.116	0.195	0.499	0.617
	0.210	0.074	0.338	3.001	0.003

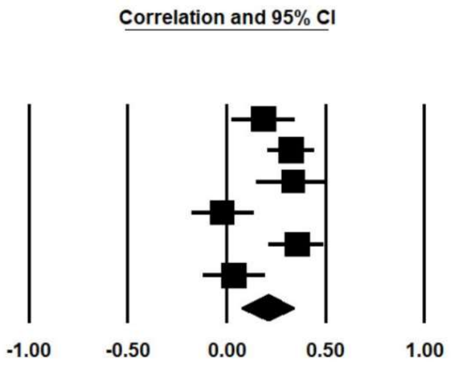

Supplementary Fig. 59. The forest plot of individual studies and the overall outcome for the genetic correlation estimate between BW-HR. Details are provided in Supplementary Fig. 1.

Study name	Correlation	Lower limit	Upper limit	Z-Value	p-Value
Mucha et al. (2014)	0.999	-1.000	1.000	0.380	0.704
Wähner et al. (2017)- Data set 1	0.190	0.157	0.223	10.964	0.000
Wähner et al. (2017)- Data set 2	0.720	0.685	0.752	25.851	0.000
Xu et al. (2011)	0.480	0.452	0.507	28.588	0.000
	0.494	0.192	0.710	3.061	0.002

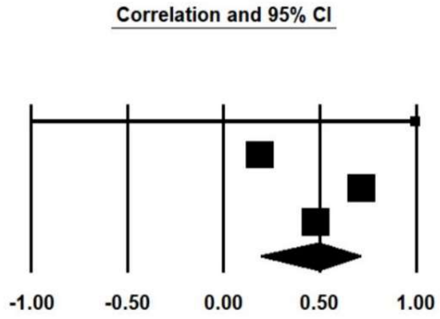

Supplementary Fig. 60. The forest plot of individual studies and the overall outcome for the genetic correlation estimate between BW-LMW. Details are provided in Supplementary Fig. 1.

Study name	Correlation	Lower limit	Upper limit	Z-Value	p-Value
Lazrul (2002)- Data set 1	0.123	-0.009	0.251	1.823	0.068
Lazrul (2002)- Data set 2	-0.088	-0.217	0.044	-1.310	0.190
Lazrul (2002)- Data set 3	-0.390	-0.580	-0.159	-3.212	0.001
Lazrul (2002)- Data set 4	-0.246	-0.394	-0.086	-2.986	0.003
Mucha et al. (2014)	0.640	0.061	0.897	2.132	0.033
	-0.078	-0.288	0.141	-0.695	0.487

(Statistics for each study)

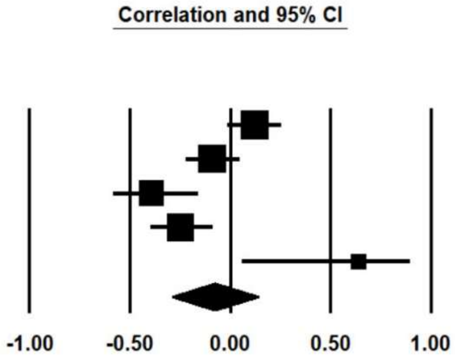

Supplementary Fig. 61. The forest plot of individual studies and the overall outcome for the genetic correlation estimate between BW-LW. Details are provided in Supplementary Fig. 1.

Study name	Correlation	Lower limit	Upper limit	Z-Value	p-Value
Drouilhet et al. (2014)	0.090	-0.565	0.676	0.242	0.809
Li et al. (2020)- Data set 1	0.430	0.167	0.636	3.092	0.002
Li et al. (2020)- Data set 2	0.220	-0.026	0.441	1.755	0.079
Zeng et al. (2018)- Data set 1	-0.120	-0.309	0.078	-1.188	0.235
Zeng et al. (2018)- Data set 2	-0.100	-0.234	0.038	-1.419	0.156
Zhang et al. (2017)	-0.040	-0.408	0.339	-0.200	0.842
	0.070	-0.124	0.260	0.708	0.479

(Statistics for each study)

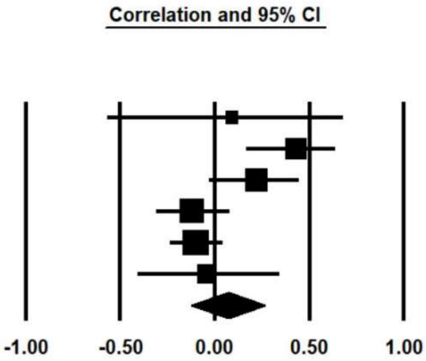

Supplementary Fig. 62. The forest plot of individual studies and the overall outcome for the genetic correlation estimate between BW-RFI. Details are provided in Supplementary Fig. 1.

Study name	Correlation	Lower limit	Upper limit	Z-Value	p-Value
Basso et al. (2012)	0.570	-0.495	0.951	1.066	0.286
Zeng et al. (2018)- Data set 1	-0.030	-0.147	0.087	-0.500	0.617
Zeng et al. (2018)- Data set 2	0.020	-0.058	0.098	0.500	0.617
	0.007	-0.059	0.072	0.197	0.844

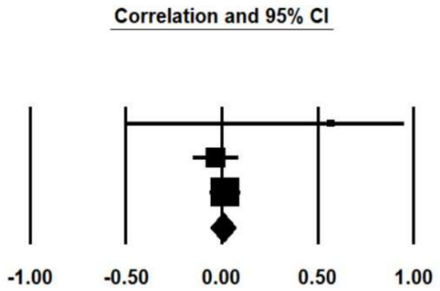

Supplementary Fig. 63. The forest plot of individual studies and the overall outcome for the genetic correlation estimate between EM-RFI. Details are provided in Supplementary Fig. 1.

Study name	Correlation	Lower limit	Upper limit	Z-Value	p-Value
Brun and Lazrul (2003)	-0.520	-0.628	-0.393	-7.008	0.000
Cheng et al. (1995)- Data set 1	-0.757	-0.851	-0.615	-7.127	0.000
Cheng et al. (1995)- Data set 2	-0.749	-0.844	-0.608	-7.169	0.000
Damayanti et al. (2019)- Data set 1	-0.300	-0.779	0.399	-0.828	0.407
Damayanti et al. (2019)- Data set 2	-0.210	-0.249	-0.170	-10.189	0.000
Hu et al. (2004)- Data set 1	-0.980	-0.993	-0.947	-9.098	0.000
Hu et al. (2004)- Data set 2	-0.960	-0.985	-0.895	-7.628	0.000
Hu et al. (2004)- Data set 3	-0.910	-0.963	-0.790	-6.565	0.000
Hu et al. (2004)- Data set 4	-0.860	-0.932	-0.724	-6.736	0.000
Lin et al. (2016)- Data set 1	-0.351	-0.824	0.410	-0.895	0.371
Lin et al. (2016)- Data set 2	-0.044	-0.659	0.606	-0.116	0.908
Lin et al. (2016)- Data set 3	0.045	-0.593	0.648	0.121	0.903
	-0.719	-0.853	-0.495	-4.902	0.000

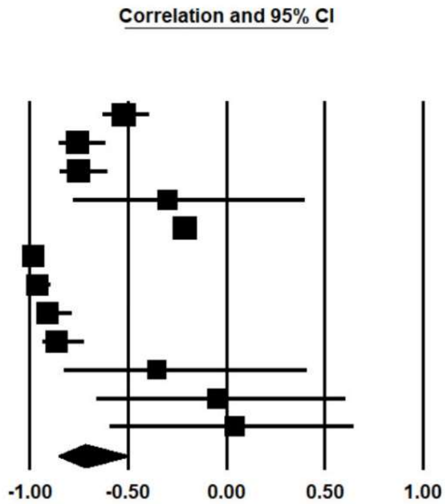

Supplementary Fig. 64. The forest plot of individual studies and the overall outcome for the genetic correlation estimate between EN-AFE. Details are provided in Supplementary Fig. 1.

Study name	Correlation	Lower limit	Upper limit	Z-Value	p-Value
El-Deghadi et al. (2022)- Data set 1	0.570	0.373	0.718	4.968	0.000
El-Deghadi et al. (2022)- Data set 2	0.780	0.733	0.820	18.608	0.000
Qiangqiang et al. (2022)	-0.690	-0.741	-0.631	-15.832	0.000
	0.273	-0.801	0.930	0.398	0.691

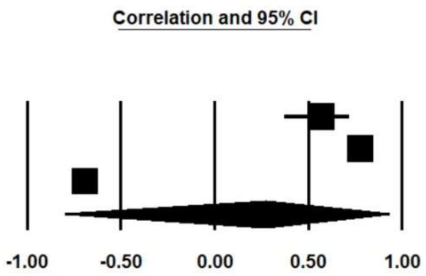

Supplementary Fig. 65. The forest plot of individual studies and the overall outcome for the genetic correlation estimate between EN-BWFE. Details are provided in Supplementary Fig. 1.

Study name	Correlation	Lower limit	Upper limit	Z-Value	p-Value
Brun and Lazrul (2003)	-0.070	-0.148	0.009	-1.744	0.081
Cheng et al. (1995)- Data set 1	0.220	0.056	0.372	2.615	0.009
Cheng et al. (1995)- Data set 2	0.322	0.159	0.468	3.761	0.000
Lin et al. (2016)- Data set 1	-0.144	-0.615	0.403	-0.497	0.620
Lin et al. (2016)- Data set 2	0.221	-0.436	0.725	0.636	0.525
Lin et al. (2016)- Data set 3	0.618	-0.223	0.932	1.492	0.136
Lin et al. (2016)- Data set 4	0.471	-0.305	0.871	1.213	0.225
	0.174	-0.044	0.376	1.564	0.118

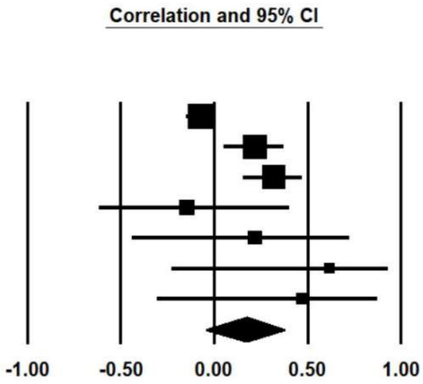

Supplementary Fig. 66. The forest plot of individual studies and the overall outcome for the genetic correlation estimate between EW-AFE. Details are provided in Supplementary Fig. 1.

Study name	Correlation	Lower limit	Upper limit	Z-Value	p-Value
El-Deghadi et al. (2022)- Data set 1	0.520	0.293	0.691	4.123	0.000
El-Deghadi et al. (2022)- Data set 2	0.770	0.661	0.847	8.838	0.000
Qiangqiang et al. (2022)	0.390	0.320	0.456	10.050	0.000
	0.580	0.269	0.781	3.357	0.001

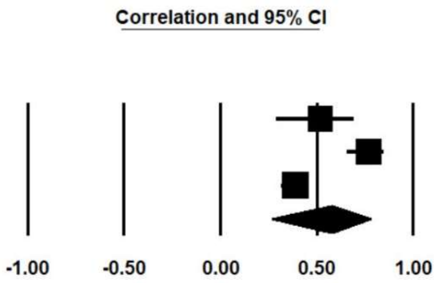

Supplementary Fig. 67. The forest plot of individual studies and the overall outcome for the genetic correlation estimate between EW-BWFE. Details are provided in Supplementary Fig. 1.

Study name	Correlation	Lower limit	Upper limit	Z-Value	p-Value
Boonlert et al. (2005)	-0.259	-0.507	0.029	-1.766	0.077
Brun and Lazrul (2003)	0.180	0.060	0.295	2.935	0.003
Cheng et al. (1995)- Data set 1	-0.214	-0.356	-0.063	-2.759	0.006
Cheng et al. (1995)- Data set 2	-0.200	-0.342	-0.049	-2.589	0.010
Cheng et al. (1995)- Data set 3	-0.354	-0.489	-0.202	-4.392	0.000
Cheng et al. (1995)- Data set 4	-0.323	-0.460	-0.172	-4.059	0.000
El-Deghadi et al. (2022)- Data set 1	0.690	0.310	0.880	3.151	0.002
El-Deghadi et al. (2022)- Data set 2	0.720	0.400	0.884	3.673	0.000
Lin et al. (2016)- Data set 1	0.189	-0.266	0.575	0.809	0.418
Lin et al. (2016)- Data set 10	-0.065	-0.474	0.368	-0.283	0.777
Lin et al. (2016)- Data set 11	0.047	-0.425	0.499	0.184	0.854
Lin et al. (2016)- Data set 12	0.112	-0.381	0.556	0.429	0.668
Lin et al. (2016)- Data set 2	0.142	-0.303	0.536	0.614	0.539
Lin et al. (2016)- Data set 3	0.191	-0.287	0.593	0.776	0.438
Lin et al. (2016)- Data set 4	0.092	-0.365	0.513	0.381	0.703
Lin et al. (2016)- Data set 5	0.020	-0.448	0.479	0.078	0.938
Lin et al. (2016)- Data set 6	0.091	-0.406	0.546	0.343	0.732
Lin et al. (2016)- Data set 7	-0.144	-0.567	0.339	-0.570	0.568
Lin et al. (2016)- Data set 8	-0.165	-0.630	0.387	-0.568	0.570
Lin et al. (2016)- Data set 9	-0.162	-0.600	0.350	-0.605	0.545
Liu et al. (2021b)	0.570	0.050	0.847	2.123	0.034
Qiangqiang et al. (2022)	0.180	-0.186	0.502	0.962	0.336
	0.030	-0.105	0.165	0.435	0.663

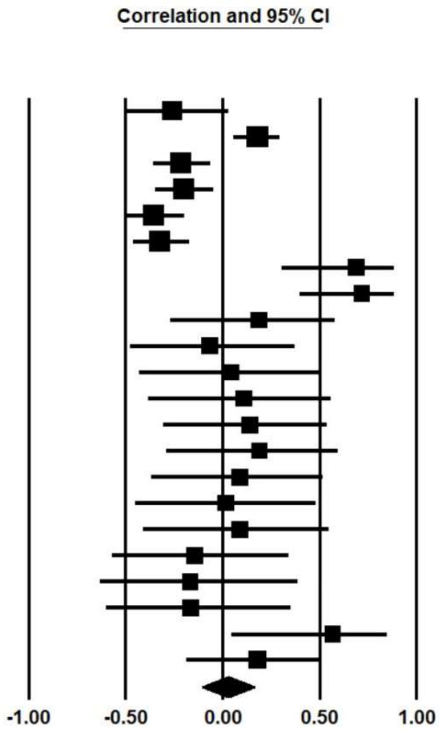

Supplementary Fig. 68. The forest plot of individual studies and the overall outcome for the genetic correlation estimate between EW-EN. Details are provided in Supplementary Fig. 1.

Study name	Correlation	Lower limit	Upper limit	Z-Value	p-Value
Chapuis et al. (2022)- Data set 1	0.060	-0.193	0.306	0.460	0.645
Chapuis et al. (2022)- Data set 2	-0.060	-0.516	0.423	-0.230	0.818
Li et al. (2020)	0.150	0.032	0.264	2.494	0.013
	0.126	0.021	0.228	2.356	0.018

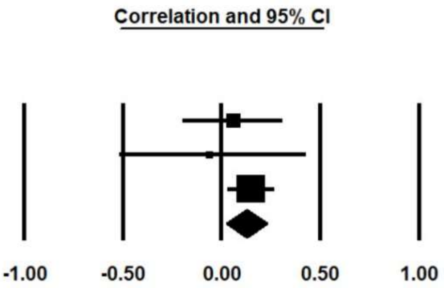

Supplementary Fig. 69. The forest plot of individual studies and the overall outcome for the genetic correlation estimate between FCR-DFR. Details are provided in Supplementary Fig. 1.

Study name	Correlation	Lower limit	Upper limit	Z-Value	p-Value
Chapuis et al. (2022)- Data set 1	0.100	-0.058	0.253	1.242	0.214
Chapuis et al. (2022)- Data set 2	0.680	0.241	0.888	2.786	0.005
Li et al. (2020)	0.120	0.048	0.191	3.260	0.001
	0.166	-0.007	0.329	1.883	0.060

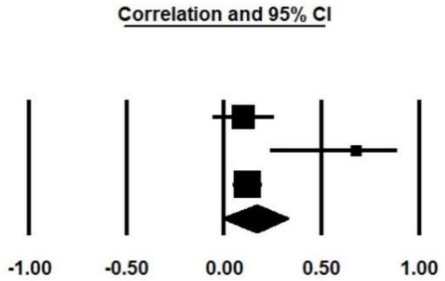

Supplementary Fig. 70. The forest plot of individual studies and the overall outcome for the genetic correlation estimate between FCR-MFI. Details are provided in Supplementary Fig. 1.

Study name	Correlation	Lower limit	Upper limit	Z-Value	p-Value
Drouilhet et al. (2014)	0.990	-1.000	1.000	0.293	0.770
Zeng et al. (2018)- Data set 1	0.470	0.107	0.722	2.484	0.013
Zeng et al. (2018)- Data set 2	0.630	0.498	0.734	7.452	0.000
Zhang et al. (2017)	0.540	0.435	0.631	8.560	0.000
	0.565	0.486	0.634	11.546	0.000

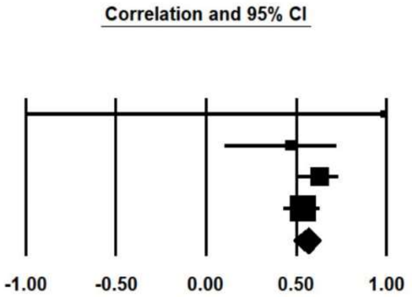

Supplementary Fig. 71. The forest plot of individual studies and the overall outcome for the genetic correlation estimate between FCR-RFI. Details are provided in Supplementary Fig. 1.

Study name	Correlation	Lower limit	Upper limit	Z-Value	p-Value
Basso et al. (2012)	-0.070	-0.760	0.694	-0.148	0.882
Marie-Etancelin et al. (2011)- Data set 1	-0.390	-0.785	0.231	-1.247	0.212
Marie-Etancelin et al. (2011)- Data set 2	0.140	-0.492	0.675	0.406	0.684
Zeng et al. (2018)- Data set 1	0.160	0.021	0.293	2.247	0.025
Zeng et al. (2018)- Data set 2	0.010	-0.049	0.069	0.333	0.739
Zhang et al. (2017)	0.490	0.148	0.727	2.716	0.007
	0.108	-0.060	0.269	1.258	0.208

(Statistics for each study)

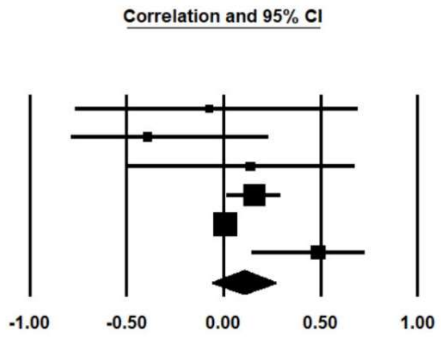

Supplementary Fig. 72. The forest plot of individual studies and the overall outcome for the genetic correlation estimate between FI-BWG. Details are provided in Supplementary Fig. 1.

Study name	Correlation	Lower limit	Upper limit	Z-Value	p-Value
Basso et al. (2012)	0.090	-0.509	0.631	0.271	0.786
Zeng et al. (2018)- Data set 1	0.670	0.303	0.864	3.191	0.001
Zeng et al. (2018)- Data set 2	0.510	0.406	0.601	8.327	0.000
	0.501	0.285	0.669	4.183	0.000

(Statistics for each study)

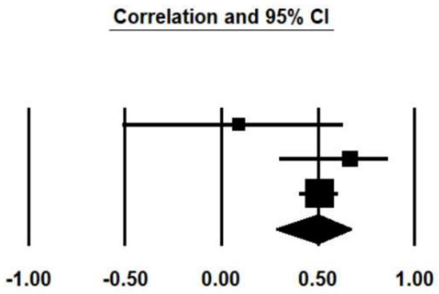

Supplementary Fig. 73. The forest plot of individual studies and the overall outcome for the genetic correlation estimate between FI-EM. Details are provided in Supplementary Fig. 1.

Study name	Correlation	Lower limit	Upper limit	Z-Value	p-Value
Chapuis et al. (2022)- Data set 1	0.330	0.079	0.542	2.546	0.011
Chapuis et al. (2022)- Data set 2	0.730	0.291	0.915	2.892	0.004
Li et al. (2020)	0.490	0.354	0.606	6.324	0.000
Liu et al. (2021b)	0.190	0.049	0.323	2.636	0.008
Zeng et al. (2018)- Data set 1	0.020	-0.193	0.231	0.182	0.856
Zeng et al. (2018)- Data set 2	0.170	-0.090	0.409	1.282	0.200
Zhang et al. (2017)	0.490	0.198	0.702	3.133	0.002
	0.318	0.150	0.468	3.625	0.000

Statistics for each study

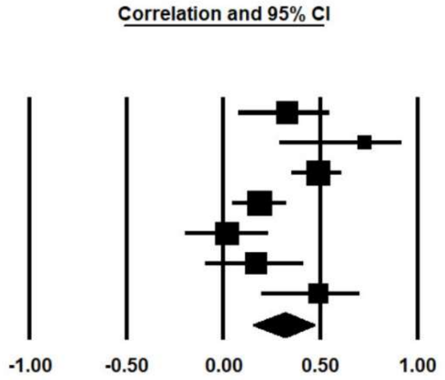

Supplementary Fig. 74. The forest plot of individual studies and the overall outcome for the genetic correlation estimate between FI-FCR. Details are provided in Supplementary Fig. 1.

Study name	Correlation	Lower limit	Upper limit	Z-Value	p-Value
Chapuis et al. (2022)- Data set 1	0.120	-0.137	0.362	0.914	0.361
Chapuis et al. (2022)- Data set 2	0.180	-0.220	0.528	0.880	0.379
Li et al. (2020)	0.170	0.068	0.269	3.245	0.001
	0.164	0.072	0.253	3.465	0.001

Statistics for each study

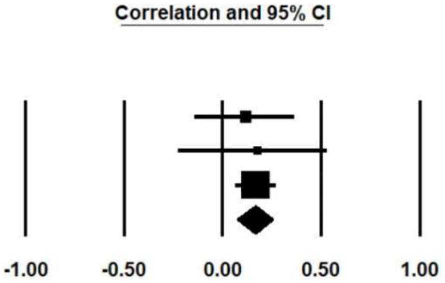

Supplementary Fig. 75. The forest plot of individual studies and the overall outcome for the genetic correlation estimate between FI-DFR. Details are provided in Supplementary Fig. 1.

Study name	Correlation	Lower limit	Upper limit	Z-Value	p-Value
Chapuis et al. (2022)- Data set 1	0.360	0.106	0.570	2.734	0.006
Chapuis et al. (2022)- Data set 2	0.690	0.441	0.840	4.442	0.000
Li et al. (2020)	0.310	0.307	0.313	194.469	0.000
	0.430	0.203	0.613	3.545	0.000

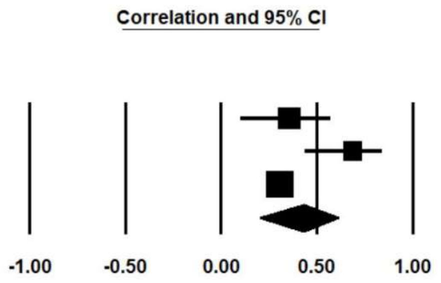

Supplementary Fig. 76. The forest plot of individual studies and the overall outcome for the genetic correlation estimate between FI-MFI. Details are provided in Supplementary Fig. 1.

Study name	Correlation	Lower limit	Upper limit	Z-Value	p-Value
Chapuis et al. (2022)- Data set 1	-0.150	-0.390	0.109	-1.136	0.256
Chapuis et al. (2022)- Data set 2	-0.200	-0.516	0.163	-1.081	0.280
Li et al. (2020)	0.050	-0.046	0.145	1.019	0.308
	-0.041	-0.203	0.122	-0.495	0.621

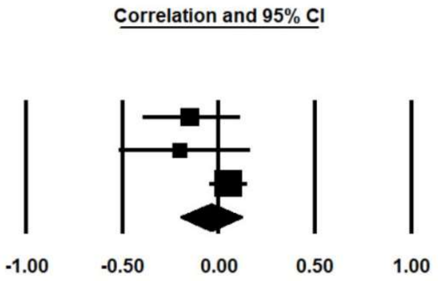

Supplementary Fig. 77. The forest plot of individual studies and the overall outcome for the genetic correlation estimate between FI-NM. Details are provided in Supplementary Fig. 1.

Study name	Correlation	Lower limit	Upper limit	Z-Value	p-Value
Basso et al. (2012)	0.890	0.642	0.969	4.223	0.000
Zeng et al. (2018)- Data set 1	0.790	0.670	0.870	8.055	0.000
Zeng et al. (2018)- Data set 2	0.860	0.758	0.921	8.420	0.000
Zhang et al. (2017)	0.770	0.199	0.951	2.443	0.015
	0.827	0.759	0.877	12.558	0.000

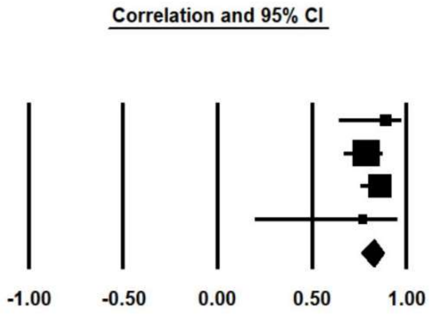

Supplementary Fig. 78. The forest plot of individual studies and the overall outcome for the genetic correlation estimate between FI-RFI. Details are provided in Supplementary Fig. 1.

Study name	Correlation	Lower limit	Upper limit	Z-Value	p-Value
	**Statistics for each study**				
Cai et al. (2023)	0.436	0.212	0.616	3.639	0.000
Mucha et al. (2014)	0.060	-0.500	0.585	0.193	0.847
Xu et al. (2011)	0.110	-0.079	0.291	1.142	0.253
	0.238	-0.036	0.479	1.706	0.088

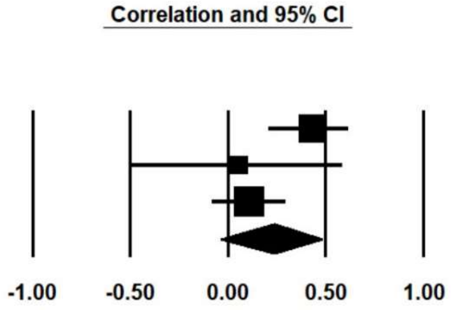

Supplementary Fig. 79. The forest plot of individual studies and the overall outcome for the genetic correlation estimate between LMW-AFW. Details are provided in Supplementary Fig. 1.

Study name	Correlation	Lower limit	Upper limit	Z-Value	p-Value
	**Statistics for each study**				
Chapuis et al. (2022)- Data set 1	0.480	0.286	0.636	4.472	0.000
Chapuis et al. (2022)- Data set 2	0.410	0.012	0.696	2.013	0.044
Li et al. (2020)	0.120	0.039	0.199	2.898	0.004
	0.323	0.022	0.571	2.099	0.036

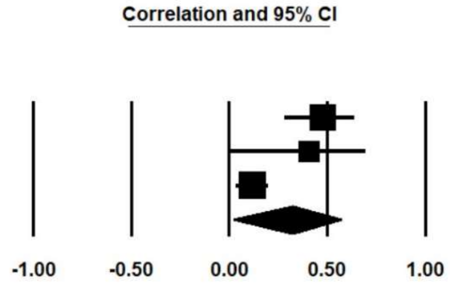

Supplementary Fig. 80. The forest plot of individual studies and the overall outcome for the genetic correlation estimate between MFI-DFR. Details are provided in Supplementary Fig. 1.

Study name	Correlation	Lower limit	Upper limit	Z-Value	p-Value
	**Statistics for each study**				
Chapuis et al. (2022)- Data set 1	-0.950	-0.998	-0.220	-2.232	0.026
Chapuis et al. (2022)- Data set 2	-0.900	-0.955	-0.786	-6.993	0.000
Li et al. (2020)	-0.930	-0.964	-0.865	-9.364	0.000
	-0.920	-0.952	-0.868	-11.875	0.000

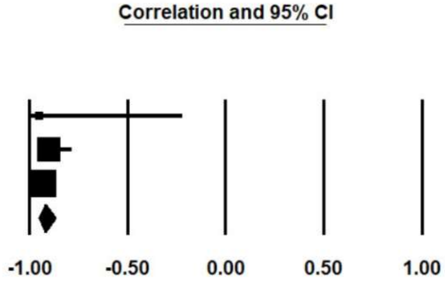

Supplementary Fig. 81. The forest plot of individual studies and the overall outcome for the genetic correlation estimate between MFI-NM. Details are provided in Supplementary Fig. 1.

Study name	Correlation	Lower limit	Upper limit	Z-Value	p-Value
Chapuis et al. (2022)- Data set 1	-0.430	-0.605	-0.216	-3.749	0.000
Chapuis et al. (2022)- Data set 2	-0.490	-0.762	-0.072	-2.263	0.024
Li et al. (2020)	0.110	0.025	0.193	2.546	0.011
	-0.261	-0.630	0.205	-1.102	0.270

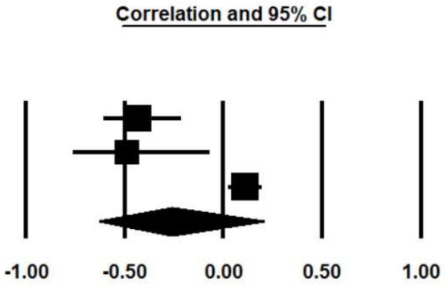

Supplementary Fig. 82. The forest plot of individual studies and the overall outcome for the genetic correlation estimate between NM-DFR. Details are provided in Supplementary Fig. 1.

Study name	Correlation	Lower limit	Upper limit	Z-Value	p-Value
Chapuis et al. (2022)- Data set 1	-0.060	-0.214	0.097	-0.748	0.454
Chapuis et al. (2022)- Data set 2	-0.440	-0.754	0.038	-1.813	0.070
Li et al. (2020)	0.060	-0.026	0.145	1.369	0.171
	-0.037	-0.206	0.133	-0.428	0.668

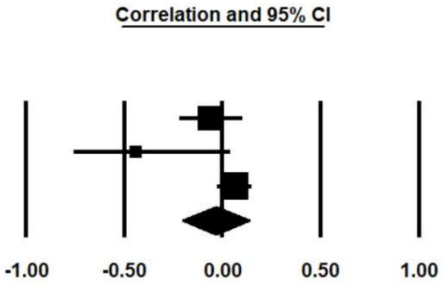

Supplementary Fig. 83. The forest plot of individual studies and the overall outcome for the genetic correlation estimate between FCR-NM. Details are provided in Supplementary Fig. 1.

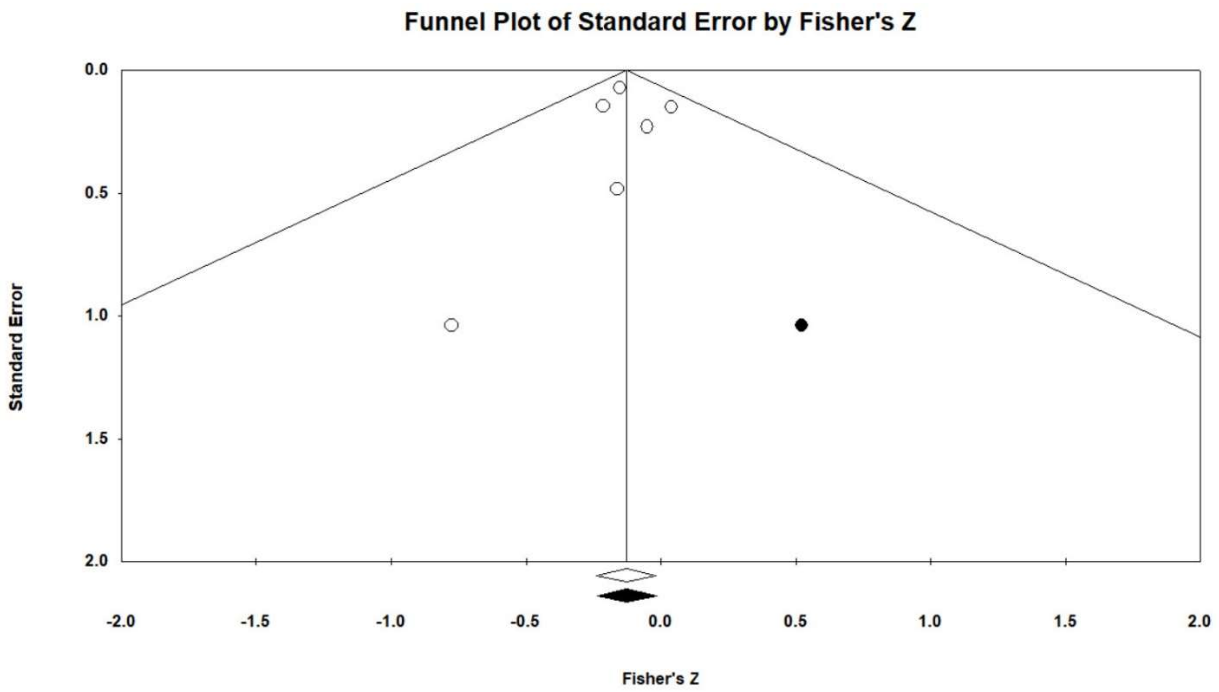

Supplementary Fig. 84. The funnel plot of the genetic correlation estimate between BWG-RFI. Details are provided in Fig. 2.

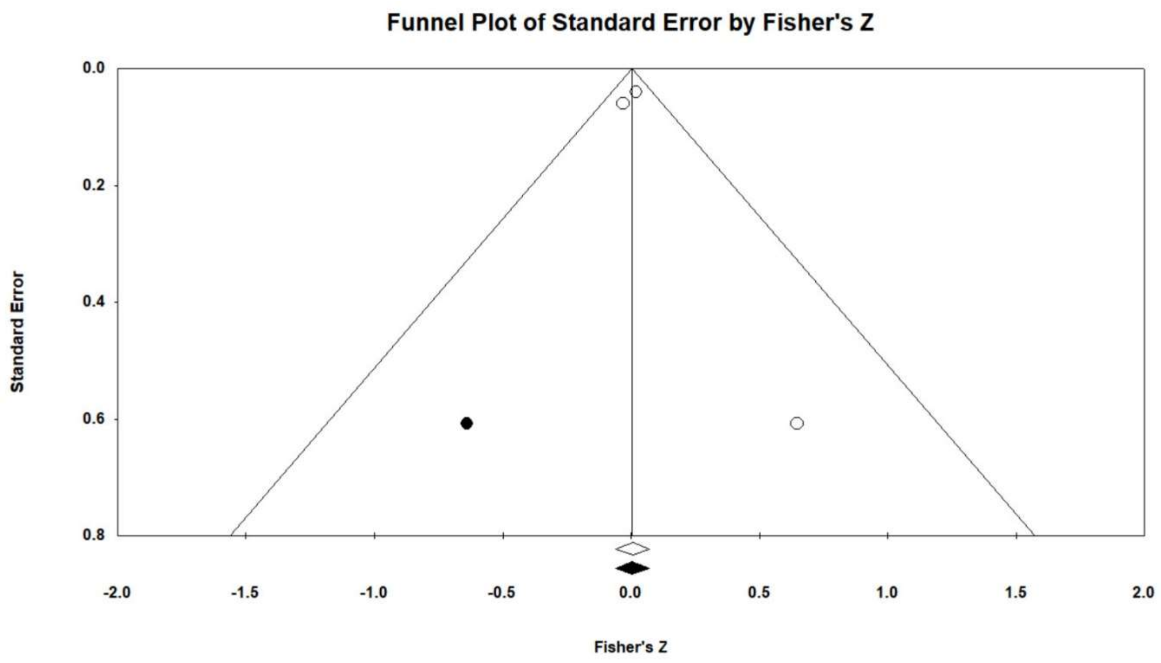

Supplementary Fig. 85. The funnel plot of the genetic correlation estimate between EM-RFI. Details are provided in Fig. 2.

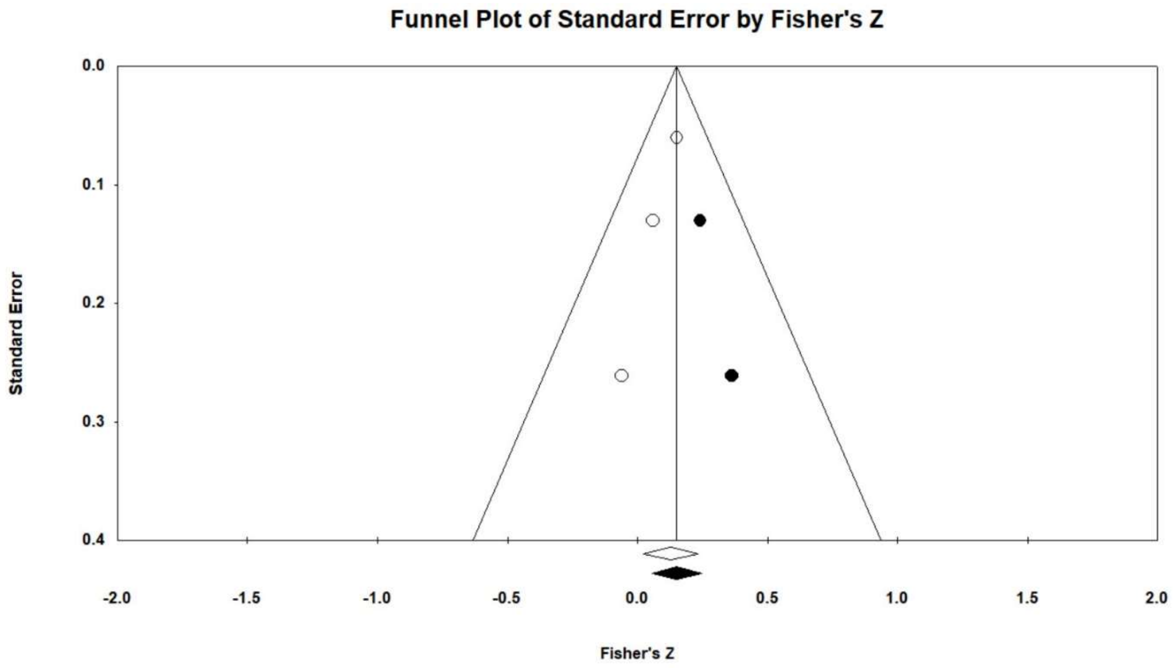

Supplementary Fig. 86. The funnel plot of the genetic correlation estimate between FCR-DFR. Details are provided in Fig. 2.

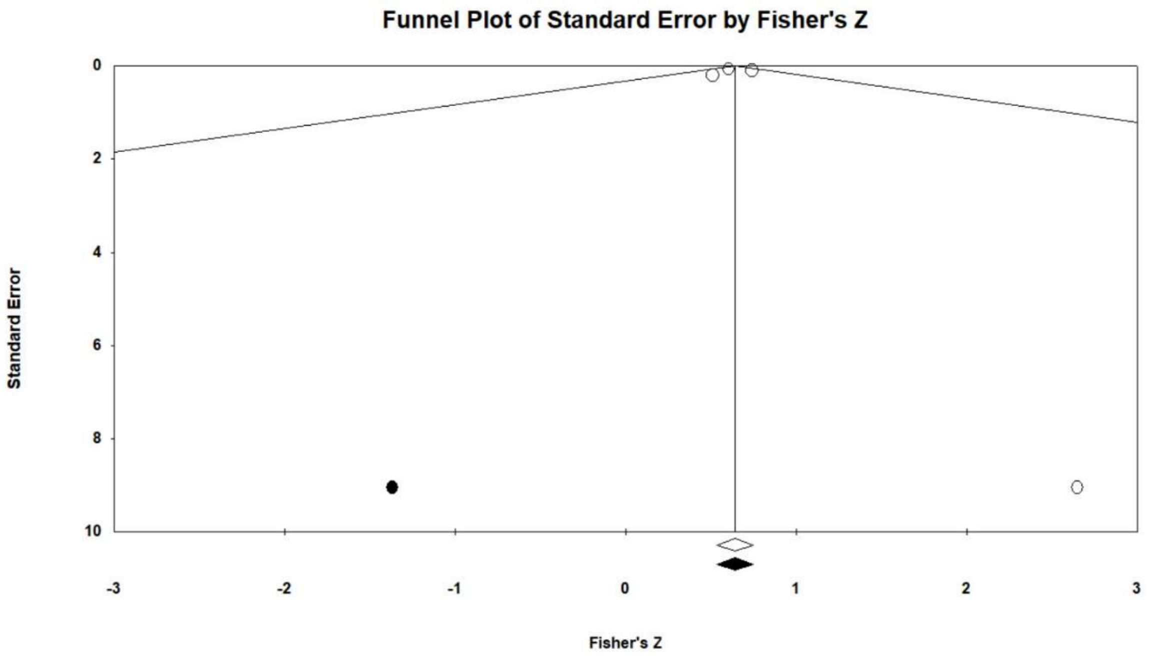

Supplementary Fig. 87. The funnel plot of the genetic correlation estimate between FCR-RFI. Details are provided in Fig. 2.

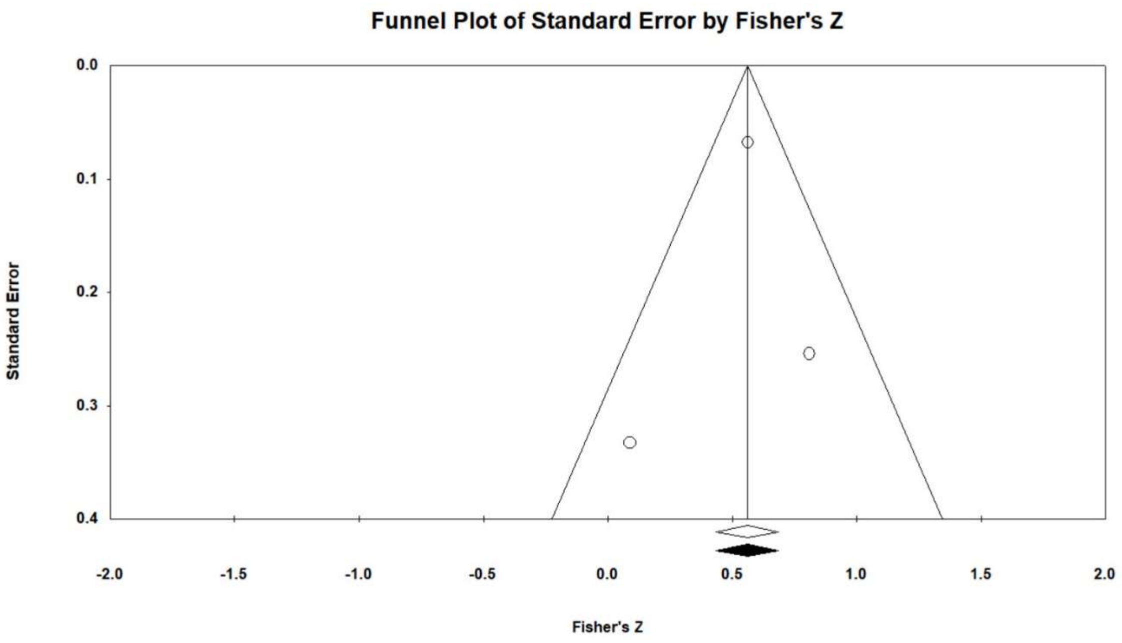

Supplementary Fig. 88. The funnel plot of the genetic correlation estimate between FI-EM. Details are provided in Fig. 2.

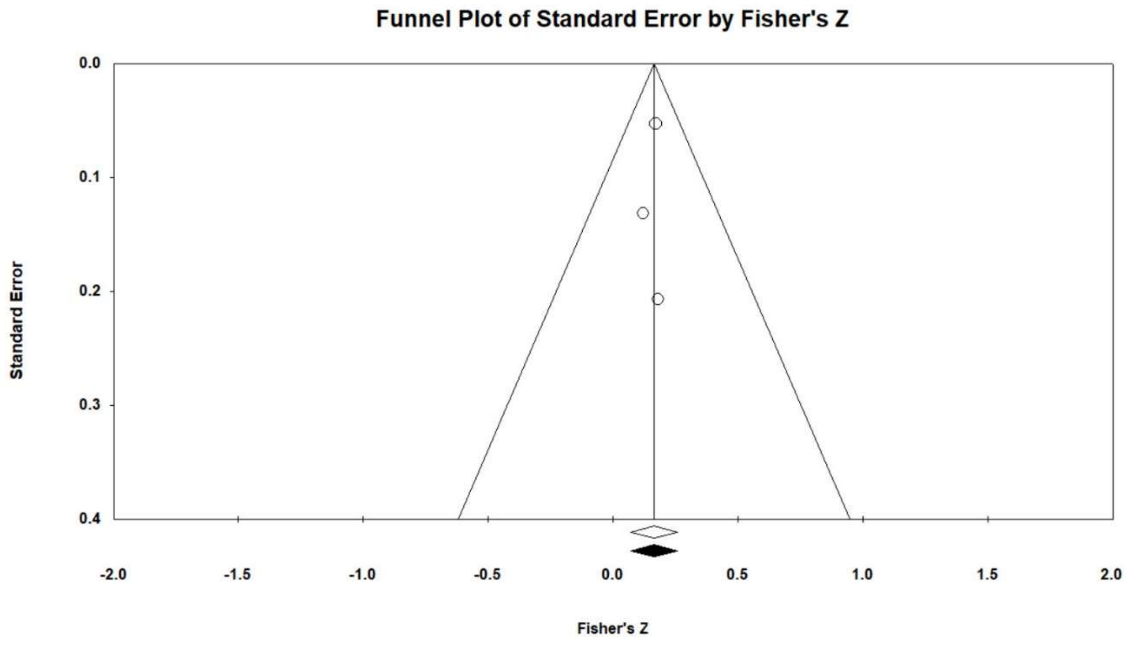

Supplementary Fig. 89. The funnel plot of the genetic correlation estimate between FI-DFR. Details are provided in Fig. 2.

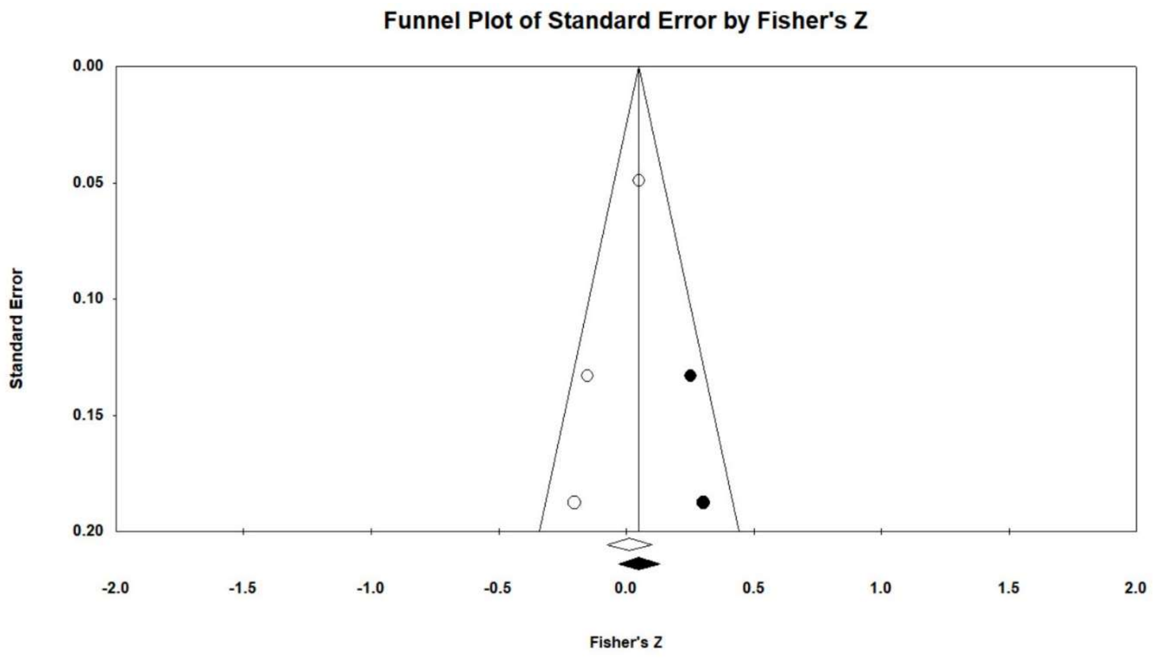

Supplementary Fig. 90. The funnel plot of the genetic correlation estimate between FI-NM. Details are provided in Fig. 2.

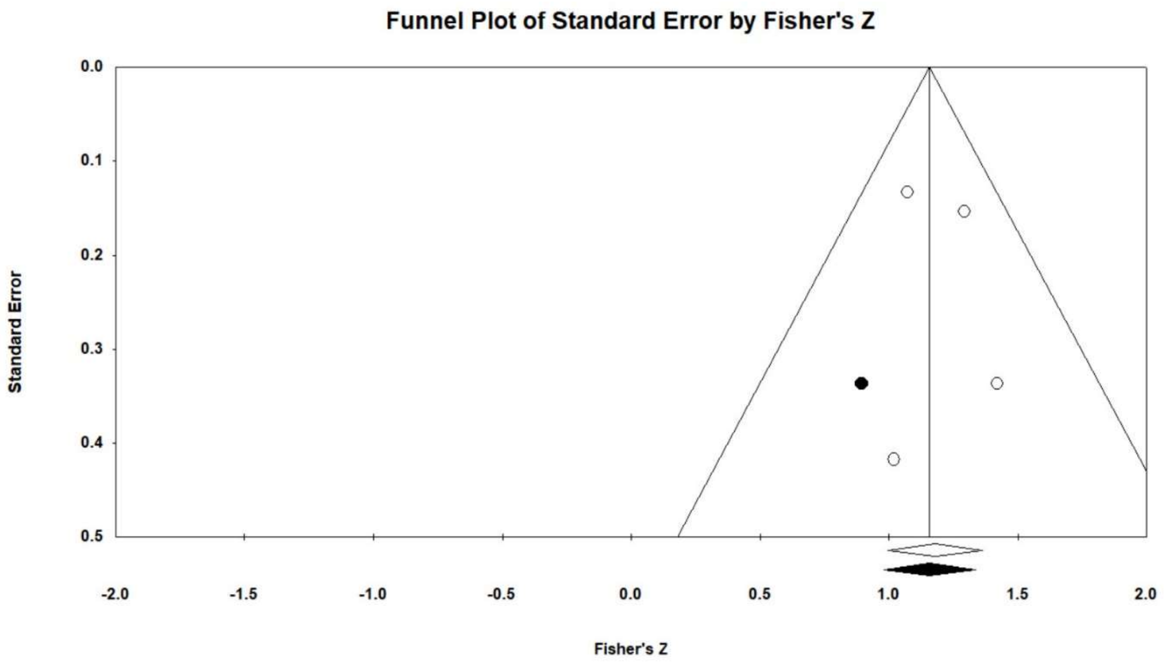

Supplementary Fig. 91. The funnel plot of the genetic correlation estimate between FI-RFI. Details are provided in Fig. 2.

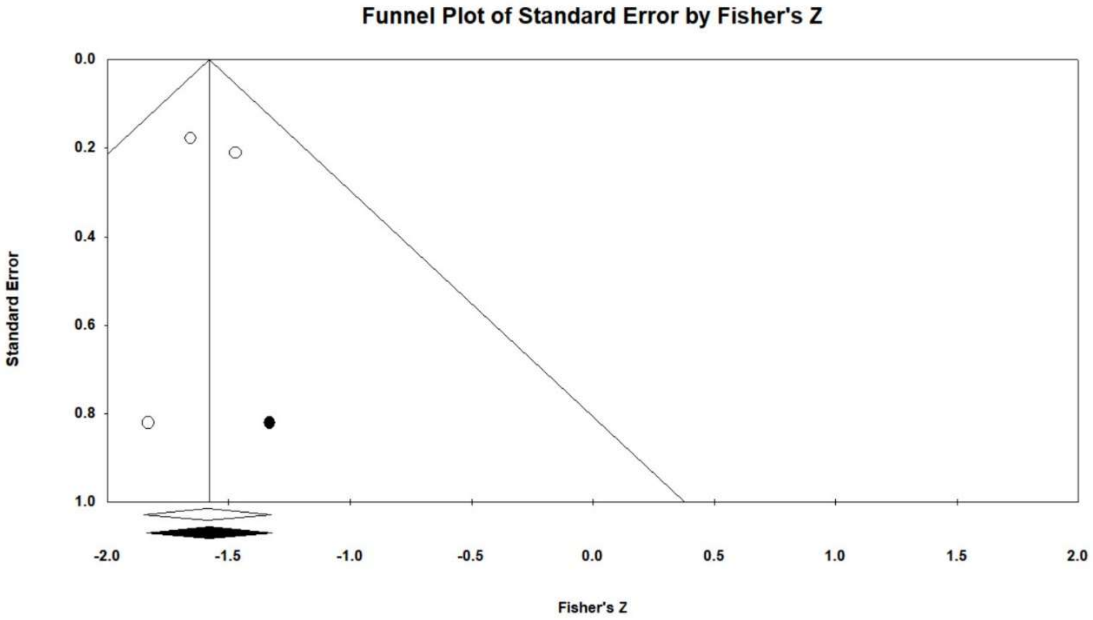

Supplementary Fig. 92. The funnel plot of the genetic correlation estimate between MFI-NM. Details are provided in Fig. 2.
